# Lignin‐Based Materials for Additive Manufacturing: Chemistry, Processing, Structures, Properties, and Applications

**DOI:** 10.1002/advs.202206055

**Published:** 2023-01-19

**Authors:** Bo Jiang, Huan Jiao, Xinyu Guo, Gegu Chen, Jiaqi Guo, Wenjuan Wu, Yongcan Jin, Guozhong Cao, Zhiqiang Liang

**Affiliations:** ^1^ Jiangsu Co‐Innovation Center of Efficient Processing and Utilization of Forest Resources International Innovation Center for Forest Chemicals and Materials Nanjing Forestry University Nanjing 210037 China; ^2^ Institute of Functional Nano & Soft Materials Laboratory (FUNSOM) Jiangsu Key Laboratory for Carbon‐Based Functional Materials & Devices Joint International Research Laboratory of Carbon‐Based Functional Materials and Devices Soochow University Suzhou 215123 China; ^3^ Beijing Key Laboratory of Lignocellulosic Chemistry Beijing Forestry University Beijing 100083 China; ^4^ Department of Materials Science and Engineering University of Washington Seattle WA 98195‐2120 USA

**Keywords:** additive manufacturing, advanced materials, engineering and functional applications, lignin, structure‐rheology relationship

## Abstract

The utilization of lignin, the most abundant aromatic biomass component, is at the forefront of sustainable engineering, energy, and environment research, where its abundance and low‐cost features enable widespread application. Constructing lignin into material parts with controlled and desired macro‐ and microstructures and properties via additive manufacturing has been recognized as a promising technology and paves the way to the practical application of lignin. Considering the rapid development and significant progress recently achieved in this field, a comprehensive and critical review and outlook on three‐dimensional (3D) printing of lignin is highly desirable. This article fulfils this demand with an overview on the structure of lignin and presents the state‐of‐the‐art of 3D printing of pristine lignin and lignin‐based composites, and highlights the key challenges. It is attempted to deliver better fundamental understanding of the impacts of morphology, microstructure, physical, chemical, and biological modifications, and composition/hybrids on the rheological behavior of lignin/polymer blends, as well as, on the mechanical, physical, and chemical performance of the 3D printed lignin‐based materials. The main points toward future developments involve hybrid manufacturing, in situ polymerization, and surface tension or energy driven molecular segregation are also elaborated and discussed to promote the high‐value utilization of lignin.

## Graphical abstract

This article provides a comprehensive view of 3D printing techniques for lignin, and presents the state‐of‐the‐art of 3D printing of pristine lignin and lignin‐based composites. Importantly, the relationship between the lignin structures and the rheological behavior of lignin/polymer blends or composites, as well as, the resulted printability in combination with the potential problems and challenges are summarized.

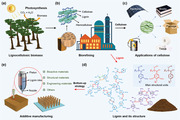

## 1 Introduction

**Figure 1:**
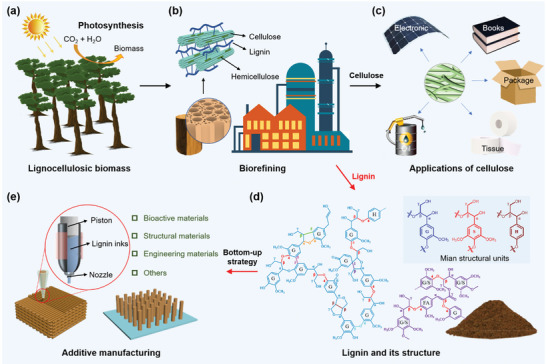
Schematic diagram of natural lignocellulosic biomass and the applications of its main constituents. a) Plant photosynthesis for demonstrating the renewable, abundant, and biodegradable features of lignocellulosic biomass. b) Biorefining of lignocellulose to separate cellulose and lignin. c) Applications of cellulose for common production and promising advanced materials. d) Morphology of the obtained lignin powder and its chemical structure. e) “Bottom‐up” additive manufacturing technology of lignin for various applications.

The ubiquity of fossil fuel‐derived non‐biodegradable plastics has resulted in serious pollution to the environment,^[^ ^1^, ^2^, ^3^ ^]^ and recent evidences indicate that the microplastics are even constantly inhaled and ingested by humans, causing a great threat to human health.^[^ ^4^, ^5^ ^]^ Exiting the era of fossil materials to a green and sustainable future requires natural abundance and renewable replacements with low or even net‐zero carbon emission. Lignocellulosic biomass, with the main constituents of cellulose, hemicellulose, and lignin, yields a great opportunity in this development due to its abundant, renewable, environmentally friendly, low‐cost, and biocompatible features (**Figure** **1**a).^[^ ^6^, ^7^, ^8^ ^]^ In which, lignin is of specific interest in this regard because it is the most abundant renewable aromatic biopolymer in nature, accounting for ≈30% of organic carbon in the biosphere. It also features many unique bioactivities or functions that are much superior to (hemi)cellulose.^[^ ^9^, ^10^, ^11^ ^]^ Lignin is generally extracted from lignocellulosic biomass by well‐established delignification methods in paper mills (Figure 1b).^[^ ^12^ ^]^ The fibrillated cellulose is often used in making papers productions for books, packages, and can also form the foundation of sustainable solutions toward a wide range of promising applications in optoelectronics, bio‐engineering, and even green energy of bioethanol (Figure 1c).^[^ ^13^, ^14^ ^]^ Unfortunately, only 2% of lignin produced from paper mills have been used for low‐value applications such as dispersants, adhesives, and additives, due to its variable chemical structure, high polydispersity in molecular weight, and rigid molecular skeleton.^[^ ^15^ ^]^ Globally, the annual output of lignin is ≈50–70 million tons, and is estimated to increase to 225 million tons by 2030.^[^ ^16^, ^17^ ^]^

Lignin mainly presents in plant cell walls, and the macromolecular structure of lignin is mainly composed of guaiacyl (G), syringyl (S), and *p*‐hydroxyphenyl (H) units that are connected via ether and carbon‐carbon bonds (Figure 1d).^[^ ^11^, ^18^ ^]^ These molecular structural features of lignin give it amphiphilicity, designable structure, and unique bioactivity, which in turn make it promising for biomedical, environmental, and engineering applications.^[^ ^19^ ^]^ However, the rigid structure of lignin often leads to high fragility to the materials. Lignin composites often exhibit difficult‐to‐tune microstructures, weak interfacial interactions, and poor stability, which greatly limit their mechanical properties and functionality. Therefore, significant challenges need to be tackled to develop advanced engineering and functional materials from lignin or lignin‐based composites for broader applications. To overcome these limitations, chemical modifications and conventional fabrication methods for lignin have been proposed to explore the unitization of lignin.^[^ ^19^, ^20^, ^21^, ^22^, ^23^, ^24^, ^25^ ^]^ However, the tedious time and complex catalytic reactions in preparing functional scaffolds and value‐added fuels by conventional methods leads to an inefficient processing. The improvement on microstructure and interfacial property of conventional lignin composites is limited due to its variable chemical structure and high polydispersity in molecular weight.^[^ ^26^ ^]^ Particularly, the limitation on constructing a stable scaffold by conventional methods impedes its high‐value applications for electronic, biomedical, and engineering materials.

More recently, additive manufacturing of lignin has attracted rapidly increasing attention due to its high applicability to various materials, high freedom in structural design and construction, rapid fabrication of tailored structures via computer aided design, tunable microstructure and properties.^[^ ^27^, ^28^, ^29^ ^]^ Additive manufacturing, also known as 3D printing, is a current state‐of‐the‐art prototyping technique and has been extensively used to build tailored engineering and functional materials for a variety of emerging applications. A variety of printing technologies have been developed to print different materials, with the most commonly used 3D printing strategies including material extrusion,^[^ ^30^ ^]^ vat photopolymerization,^[^ ^31^ ^]^ powder bed fusion,^[^ ^32^ ^]^ material and binder jetting,^[^ ^33^ ^]^ sheet lamination,^[^ ^34^ ^]^ and direct‐energy deposition.^[^ ^35^ ^]^ Over the last decade, 3D printing has been employed to handle a broad range of materials, including polymers, ceramics, metals, and semiconductors, for electronics, robotics, energy storage, organs, satellites, etc.^[^ ^36^, ^37^, ^38^, ^39^, ^40^ ^]^ In 3D printing, the high‐degree‐of‐freedom robot arm can drive the print‐head/nozzle to move flexibly in 3D space to the coordinates set by the computer, thus enabling the rapid preparation of product with complex geometry. Importantly, for fused filament fabrication (FFF) and direct ink writing (DIW) printing models, the shear‐force generated when the ink is extruded from the fine nozzle enables fillers in the ink that have asymmetrical shapes to align along the filaments, which can greatly improve the mechanical, charge and thermal transport performance of printed structures.^[^ ^41^, ^42^ ^]^

As an abundant and renewable biopolymer, the use of 3D printing technologies to print lignin into composite materials with desired mechanical and functional properties would make it an extremely promising alternative to fossil‐based polymers such as acrylonitrile butadiene styrene (ABS), polyamide, polycarbonate, and epoxy resins. The macro‐ and microstructures, and the resulting properties of 3D printed lignin composites can be tuned to meet the requirements of optics, energy, drug delivery, and personalized medicine (Figure 1e). The first research combining printing technology and lignin to construct 3D lignin‐based materials was reported in 2010s.^[^ ^43^ ^]^ Since then, 3D printing of lignin has flourished and greatly broaden the application areas of lignin‐based materials, which is at the frontier of lignin research. Although rapid progress in the 3D‐printing of lignin has been achieved,^[^ ^44^, ^45^ ^]^ many challenges, such as the unclear structure‐rheological relationship and limited applications, need to be tackled for the broad adoption of 3D printed lignin composites. Although the modification of lignin and its physical and chemical properties have been reviewed in several excellent papers,^[^ ^10^, ^11^, ^12^, ^15^, ^19^, ^20^, ^21^ ^]^ there are limited comprehensive reviews and outlook on 3D printing of lignin (especially the structure‐rheology relationship), and the properties and applications of printed materials.

This paper reviews recent advances in 3D printing of lignin and their applications to offer perspectives for the future development. The molecular structure and properties of lignin in different species or tissues of plants, and the effect of physical, chemical, and biological treatments on lignin structure are briefly discussed. Then, we provide a comprehensive view of 3D printing techniques for lignin, mainly including the working principles of representative 3D printers of FFF, DIW, stereolithography (SLA), and selective laser sintering (SLS). Importantly, the relationship between the lignin structures and the rheological behavior of lignin/polymer blends or composites, as well as the resulted printability in combination with the potential problems and challenges are summarized. We also highlight the key issues toward future developments in the 3D printing of lignin, and aim to promote the high‐value utilization of lignin in various engineering and functional materials. This review may help to ascertain the structure‐rheology relationship of lignin or lignin/polymer blends, the challenges and emerging techniques in using lignin for 3D printing, and to promote the practical application of lignin.

## 2 Structure and Property of Lignin

Lignin is a natural biopolymer in lignocellulosic biomass with the main constituents of G, S, and H units linked by carbon‐carbon and ether bonds.^[^ ^11^, ^18^, ^46^ ^]^ As the only large‐volume renewable biopolymer that consists of aromatics, lignin is derived from the radical polymerization of substituted phenylpropane units and starts with the deamination of phenylalanine.^[^ ^18^, ^47^, ^48^, ^49^ ^]^ Then the formed cinnamic acid is converted into the three monomers of *p*‐coumaryl, coniferyl, and sinapyl alcohols under a series of oxidases and peroxidases processes, such as caffeic acid *O*‐methyltransferase, cinnamyl alcohol dehydrogenase, and ferulate 5‐hydroxylase. The aromatic monomers are further transferred and polymerized in vivo through an enzyme‐mediated dehydrogenation polymerization, resulting in a cross‐linked amorphous lignin structure. This biosynthesis pathway involves successive reactions of hydroxylation, methylation, reduction, and radical coupling, leading to the complex structural features of lignin.

With various biosynthetic enzymes, the content and structural constituents of lignin in woody (softwood and hardwood) and gramineous resources are rather different from each other.^[^ ^50^, ^51^ ^]^ The relative amounts of lignin in wood are generally much higher than those in gramineous plants, with lignin content in pine,^[^ ^52^, ^53^, ^54^ ^]^ eucalyptus,^[^ ^55^ ^]^ and poplar^[^ ^54^, ^56^ ^]^ accounting for ≈28–31%, 25–29%, 21–27%, respectively, and that in rice straw,^[^ ^57^ ^]^ corn stover,^[^ ^58^ ^]^ switchgrass,^[^ ^59^ ^]^ and miscanthus^[^ ^60^ ^]^ accounting for ≈20%, ≈18%, 17–18%, and 20–24.5%, respectively. For normal wood, lignin mainly presents in the secondary cell walls of plants, especially in the middle layer, but the external environment is also an important issue affecting the lignin content and distribution. For tension wood, namely the xylem is suffered tensile strength by the sloping or curved trunk and branches, the secondary cell walls are mainly composed of highly crystalline cellulose, and the content of lignin decreases when compared with that in a normal one.^[^ ^18^, ^61^ ^]^ In contrast, the middle layer of secondary cell walls in compression wood has higher lignin content than that in normal wood due to its high degree of lignification.^[^ ^61^ ^]^

**Figure 2:**
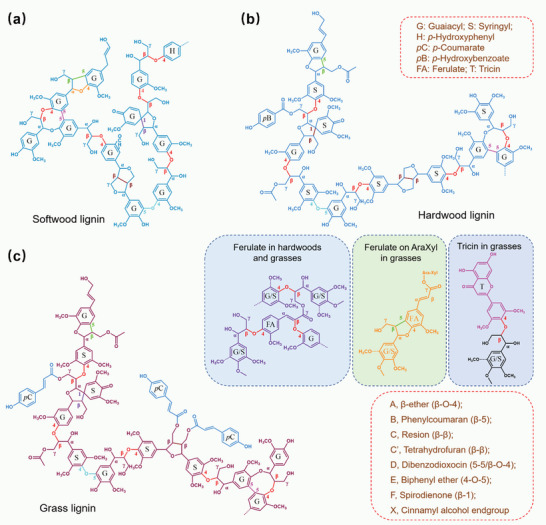
Structure and properties of lignin extracted from a) softwood, b) hardwood, and c) grass. Lignin in softwood is mainly composed of G units. The hardwood lignin mainly consists of G and S units with minor *p*‐hydroxybenzoate and ferulates. The grass lignin contains all G, S, and H units, and *p*‐coumarates, ferulates, and tricin are also present in grass lignin. The main linkages of lignin involve *β*‐O‐4, 4‐O‐5, *β*‐1, and *β*‐*β*, but show huge differences in content, stereostructure, and properties in different plants. Reproduced with permission.^[^ ^49^ ^]^ Copyright 2019, Elsevier.

The structural features of lignin in woody and gramineous resources also exhibit a huge difference (**Figure** **2**).^[^ ^11^, ^18^, ^51^ ^]^ Gymnosperm lignin lacks S units and is mainly composed of G units (Figure 2a). As a result, gymnosperm lignin is generally more branched than the typical G/S‐rich angiosperm lignin (Figure 2b). Gramineous lignin contains all structural units of G, S, and H, meanwhile, *p*‐coumarates and ferulates are also abundant in the cell walls of gramineous lignin and are chemically linked with phenylpropane structures (Figure 2c). In addition, tricin is discovered in almost all gramineous lignins such as bamboo,^[^ ^62^ ^]^ wheat,^[^ ^63^, ^64^ ^]^ rice,^[^ ^65^, ^66^ ^]^ and vanilla (aerial roots),^[^ ^67^ ^]^ and is considered to be an authentic lignin monomer due to its being fully compatible with the process of lignification. It has been reported that tricin links to lignin mainly via *β*‐O‐4 linkages,^[^ ^64^, ^68^, ^69^ ^]^ which makes lignin in gramineous plants more complex than that in wood.

The S/G ratio is generally used to describe the structural features of lignin, and compared with angiosperm lignin, where the S/G ratio is generally over 1.5, gramineous lignin often shows lower S/G ratio (0.8).^[^ ^70^ ^]^ Although lignin is generally dominant in *β*‐O‐4 linkages, typically accounting for over 50%, followed by the *β*‐5, *β*‐1, *β*‐*β*, 5‐5, and 4‐O‐5 bonds, their contents in various plants exhibit a huge difference. With abundant G units in gymnosperm lignin, the active C_5_ position leads to a much higher condensation degree than angiosperm lignin, endowing gymnosperm lignin with stronger recalcitrance. Angiosperm lignin is rich in *β*‐O‐4 linkages and shows a lower condensation degree.^[^ ^71^, ^72^ ^]^ In addition, more structural units in angiosperm lignin are cross‐linked to polysaccharides. In detail, lignin is mainly bonded with feruloylated xylans by radical coupling or nucleophilic addition,^[^ ^18^, ^73^, ^74^ ^]^ yielding much ether‐linked lignin‐carbohydrate complex, as a result, angiosperm lignin shows excellent viscoelasticity.

The structure of lignin in different plant tissues also displays a significant difference. With wheat stems and leaves as an example,^[^ ^75^, ^76^ ^]^ it was found that lignin content in wheat straw stems (23.8%) was higher than that in wheat straw leaves (16.6%). The results of structural characterization reveal that stem lignin shows more abundant S units and *β*‐O‐4 aryl ether bonds than leaf lignin. Consistent with the above results, the difference in nitrobenzene oxidation yield between leaf lignin (1.60 mmol g^−1^) and stem lignin (2.23 mmol g^−1^) suggests that the condensation degree of leaf lignin is much higher than that of stem lignin. In stereochemistry, the side chain parts of lignin also have different conformations. By ozonation treatment, the aromatic parts and double bonds of lignin can be destroyed completely, and the side chain parts of lignin will be converted into simple organic acids, but with the original stereo structure of the side chain kept intact.^[^ ^77^, ^78^, ^79^ ^]^ This method is often used to obtain the information of stereo configuration of the side chain especially the *β*‐O‐4 aryl ether bonds in lignin. The stereo chemical configuration of the lignin side chain is often divided into erythron (E) and threo (T) types, in which the E and T orms of *β*‐aryl ethers yield erythronic acid and threonic acid, respectively. By comparison, it reported that the E types are dominant in wheat stem and leaf lignin, with the results consistent with that in hardwood lignin but different from that in softwood lignin. In addition, the wheat stem lignin shows a much higher E/T ratio than leaf lignin, and the result is also consistent with lignin in rice stems and leaves, that is mainly governed by the S/G ratio.

The physical, chemical, and biological treatments can significantly alter the structure of lignin. The frequently used commercial lignins mainly come from paper or biorefinery mills as by‐products, such as soda, kraft, and organosolv lignins (OSLs). Depolymerization and condensation of lignin generally occur during physical, chemical, and biological treatments, which may change its molecular weight, polydispersity, linkages, and functional groups.^[^ ^9^, ^10^, ^11^, ^12^ ^]^ Bioengineering to modify lignin or incorporate typical components is often conducted to increase the functionalization of lignin, such as grafting copolymers and synthesizing new chemically active sites.^[^ ^19^ ^]^ The unique structural features endow lignin with natural bioactivity, hydrophilicity, and hydrophobicity, nano‐scale adjustability, flexibility in structural modification, and biocompatibility. Based on these distinct advantages, recent achievements have been achieved in the application research of lignin. It has been demonstrated that lignin can be directly used as a natural antioxidant, UV protectant, antibacterial agent, and binder.^[^ ^46^, ^51^, ^80^ ^]^ Moreover, nanomaterials, gel composites, and responsive smart materials, and carbon‐based materials further broaden the functions of lignin, which offer a great potential for advanced materials.^[^ ^10^, ^81^, ^82^, ^83^ ^]^ For example, with redox structure, lignin exhibits great potential in energy storage.^[^ ^84^, ^85^ ^]^ Therefore, constructing lignin into structures with desired macro‐ and microstructures and properties by a facile and cost‐efficient method can greatly promote the practical application of lignin. Additive manufacturing as a facile and scalable technique is a promising way to add value to lignin. Considerable efforts have been devoted to developing 3D printing of lignin, and the printing of lignin and its composites with different printing technology routes such as FFF, DIW, SLA, and SLS has been achieved. However, the unclear structure‐rheology relationship and limited applications need to be tackled for the broad adoption of 3D printed lignin composites. Therefore, a comprehensive review of additive manufacturing technologies for lignin and the structure, properties and applications of printed lignin materials is highly desirable, which helps to change the traditional concept of “one can make anything from lignin except money” to “one can make anything from lignin include money”.

## 3 3D Printing Technique for Lignin

The microstructures and properties of printed polymer composites are influenced by the printing parameters (e.g., temperature, nozzle diameters, and printing speed), and in turn, the molecular structure and viscoelasticity of polymers largely affect the whole printing process. Therefore, understanding the structure‐process‐property relationships in 3D printing is critical for constructing tailored objects with designed structure and property. Each 3D printing process has unique characteristics and advantages in the fabrication of polymer materials. Likewise, 3D printing requires specific conditions of the polymers, such as the state (solid filament, liquid, and/or powder) and rheological properties (modulus and viscosity). Although in theory polymers involved lignin are supposed to be appropriate for almost all types of 3D printing techniques, progress in recent years demonstrates that lignin is only successful for techniques of material extrusion (FFF and DIW), SLA, and SLS due to its complex and unique structure.

### 3.1 Fused Filament Fabrication

**Figure 3:**
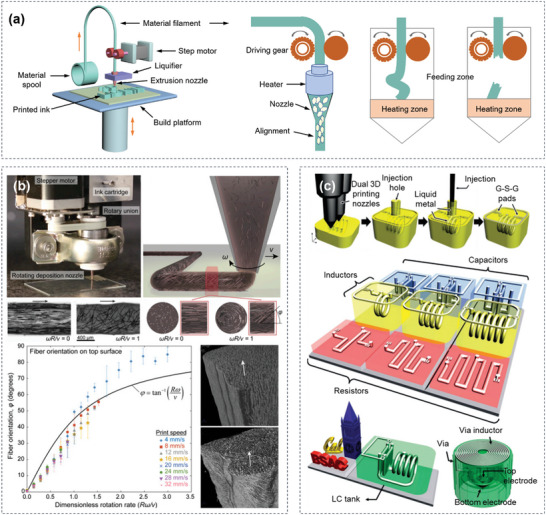
Schematic of FFF 3D printing techniques and the applications of printed materials. a) Schematically showing the main components of a FFF printer, the orientation of printed composition, and the effects of filament loading on printing behaviors including buckling and fracture. b) Rotational FFF printing. The rotation angle of the nozzle is controlled by a 3D motion‐control system and the schematic view or optical micrographs of fiber orientation with different helical angles affected by the rotational rate (*ω*) and translational velocity (*v*). The relationship between the dimensionless rotation rate (*Rω*/*v*) and fiber orientation (*φ*), as well as, the cross‐sectional views of printed filaments to demonstrate the rotation of the internal structure. Reproduced with permission.^[^ ^96^ ^]^ Copyright 2018, National Academy of Sciences. c) Schematic of the 3D‐printed microelectronics for integrated circuitry and passive wireless sensors, including the fabrication process, the microelectronics components of capacitors, inductors, and resistors, and an inductor‐capacitor tank, as well as the wireless passive sensor mainly composed of electrodes and spiral inductor. Reproduced with permission.^[^ ^102^ ^]^ Copyright 2015, Spronger Nature.

FFF, also known as melt‐extrusion 3D printing, is a popular method for handling polymers and polymer‐based composites.^[^ ^86^, ^87^, ^88^ ^]^ It is a simple and low cost ($300 for home use and $2–8 k for professional use) technique with a wide range of printing rates (50–500 mm h^−1^).^[^ ^40^ ^]^ Generally, a solid polymer monofilament with a diameter of ≈0.5–3 mm is squeezed into a heated chamber by a step‐motor where the polymer melts, and then extruded out through an extrusion nozzle with a preset temperature (depending on its melt rheology, **Figure** **3**a). Preferably, the temperature of the heated chamber should slightly above the glass‐transition temperature (*T*_g_) of the polymer to obtain favorable melt rheology. The solid polymer filament acts as not only a feedstock but also a piston that pushes the molten mass into the hot nozzle. Controlled by a preset program, the molten polymer is further extruded to the x‐y plane and gradually solidifies on a platform to form a 3D pattern though layer‐by‐layer deposition.^[^ ^89^, ^90^, ^91^ ^]^ Thermoplastic materials such as polylactic acid (PLA), nylon, and ABS, elastomers such as polyetheresters are often used as feedstocks for FFF due to their low melting temperature and appropriate melt rheology.^[^ ^40^, ^92^ ^]^ For a particular filament, the internal diameter of the extrusion nozzle, build speed, shape, and processing temperature are vital parameters for a successful build process. Many studies have sought to address the effect of FFF process parameters on the surface microstructure, dimensional accuracy, mechanical properties, pore structure of printed polymer. The printing parameters such as temperature gradients, feed rate, die swelling, and even addition of stabilizers and other additives have also been investigated to improve the efficiency in the FFF process.^[^ ^86^, ^87^, ^88^, ^89^, ^90^, ^91^, ^92^ ^]^ It has been demonstrated that the ratio of modulus/melt viscosity (*E*/*ƞ*) of the feedstock has an important effect on the buckling property of a printed object, which will not occur if *E*/*ƞ* is above a critical range of 3 × 10^5^–5 × 10^5^ s^−1^.^[^ ^93^, ^94^ ^]^ Therefore, the polymer filament should display a minimal stiffness before entering the heated zone. Induced by the generated shear stress, the polymer chain or anisotropic lamellar structure tends to be aligned along the printing direction, where the printing rate and viscoelasticity of the molten polymer play an important role.^[^ ^95^, ^96^ ^]^ As a result, the mechanical strength is highly improved along the chain direction, much superior to that of the transverse direction. However, the solid polymer filament also acts as a piston, once the filament loading exceeds the limit rate, shear failure often occurs between the pinch roller and the drive wheels (Figure 3a).^[^ ^97^ ^]^ For continuous and stable printing, some water‐soluble additives, such as polyvinyl alcohol (PVA) and sacrificial molds or templates, are commonly used to improve the melt rheology for FFF or act as temporary support structures, which can be easily removed by immersion in water in post‐processing.^[^ ^98^, ^99^, ^100^, ^101^ ^]^

With tunable structures and functions, materials printed by FFF have been widely used in electronics, energy storage, and bio‐tissues. Raney et al.^[^ ^96^ ^]^ developed a rotational FFF 3D printing strategy that enables spatially controlled orientation of short carbon fibers (CF) in epoxy matrix (Figure 3b). By controlling the nozzle rotation speed and the printing speed, the resulting CF‐epoxy composites display optimized mechanical strength and damage tolerance, which broadens the design of fiber arrangements, microstructure adjustability, and performance space for fiber‐reinforced composites. FFF has also been employed for high‐resolution multiple‐nozzle 3D printing. Wu et al.^[^ ^102^ ^]^ designed and constructed a functional 3D structure using a two‐nozzle 3D printing system with a printing resolution of 30 µm for integrated circuitry and passive wireless sensors. By using the FFF technology and a multiple‐nozzle system, 3D scaffold with both supporting and sacrificial structures is constructed. After removing the sacrificial materials, liquid metal paste is injected and subsequently solidified to form conductive electrical structures (Figure 3c). It establishes an innovative approach to fabricate a 3D integrated circuitry with embedded electrical structures for various applications. However, not all molten polymers exhibit efficient printability. It has been demonstrated that the poor homogeneity of molecular chains, high melt viscosity, and wide polydispersity often cause nozzle clogging and suboptimal quality of manufactured products.^[^ ^38^ ^]^ Therefore, the adaptability between FFF printers and feedstocks is critical to smoothly printing objects with desired micro‐ and macro‐structures.

### 3.2 Direct Ink Writing

**Figure 4:**
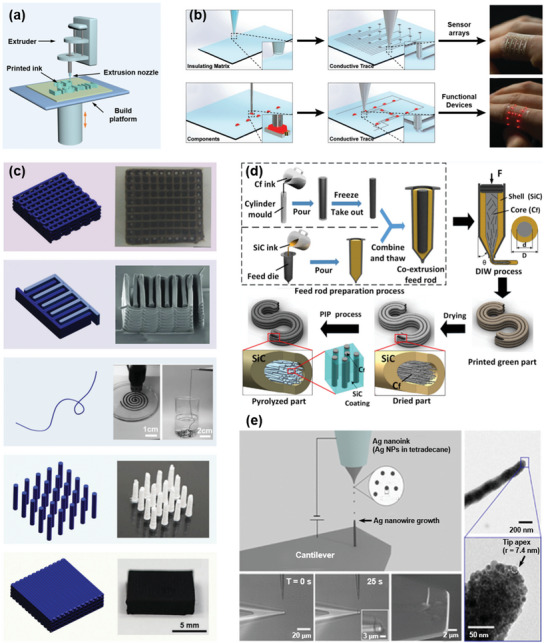
Schematic of DIW 3D printing technique and the applications of printed materials. a) Schematically showing the main structure of a DIW printer. b) Hybrid 3D printing for soft electronics, including the schematic or optical images to show the DIW process of insulating matrix and conductive trace, as well as the pick‐and‐place process of components to form a sensor array or functional device for soft materials applied to the human finger. Reproduced with permission.^[^ ^117^ ^]^ Copyright 2017, Wiley‐VCH. c) Different structural and functional designs for DIW, including gridding, interdigitated sharp, vertical pillars, and compact structure. From top to bottom: Reproduced with permission.^[^ ^121^, ^124^, ^125^, ^126^, ^127^ ^]^ Reproduced with permission.^[^ ^124^ ^]^ Copyright 2018, Weily‐VCH. Reproduced with permission.^[^ ^125^ ^]^ Copyright 2013, Wiley‐VCH. Reproduced with permission.^[^ ^126^ ^]^ Copyright 2017, Wiley‐VCH. Reproduced with permission.^[^ ^121^ ^]^ Copyright 2019, American Chemical Society. Reproduced with permission.^[^ ^127^ ^]^ Copyright 2019, Wiley‐VCH.   d) DIW printing of a core–shell carbon fiber/silicon carbide composite to provide high strength and toughness to the material. The ink‐induced yield tress contributes the high orientation, thus enhancing the material toughness while the dense silicon carbide shell contributes to the strength. Reproduced with permission.^[^ ^128^ ^]^ Copyright 2019, Elsevier. e) Direct printing of high‐aspect‐ratio atomic force microscopy probe and its optical micrographs. Electrohydrodynamic printing of a freestanding Ag nanowire directly on a tipless silicon nitride cantilever. Reproduced with permission.^[^ ^144^ ^]^

DIW shows distinct advantages of excellent material compatibility, high spatial resolution, and low‐cost, which constructs arbitrary 3D structures through a layer‐by‐layer ink‐filaments deposition subsequence.^[^ ^103^, ^104^, ^105^, ^106^, ^107^, ^108^ ^]^ DIW 3D printing technology includes the following two strategies: 1) Extrusion‐based approach and 2) meniscus‐guided approach, where the resolution of extrusion‐based approach can reach 1 µm,^[^ ^109^ ^]^ and that of another one is even as high as 600 nm.^[^ ^110^ ^]^ For extrusion‐based DIW, a viscoelastic ink is extruded through a fine nozzle under adequate printing pressures with the pattern‐generating program (flow rate and shape) controlled by a computer (**Figure** **4**a). The extrusion‐based DIW process can be further divided into filamentary‐based and droplet‐based approaches. The filamentary‐based approaches mainly include robocasting (or robotic deposition)^[^ ^111^, ^112^ ^]^ and micropen direct writing.^[^ ^113^ ^]^ The droplet‐based approaches include ink‐jet^[^ ^114^, ^115^ ^]^ and hot‐melt ink^[^ ^116^ ^]^ printing. It reveals that the ink designs have a critical influence on material printability. The development of a printable ink with appropriate rheological properties is the most important part for DIW, and they directly affect the resolution and properties of the printed materials. By carefully controlling the rheological behavior, inks of colloidal suspensions, colloidal gels or dilute fluids, polymer liquids, hydrogels, waxes, metals or metal oxides, semiconductors, polyelectrolyte complexes, etc., have been successfully employed to make them available for DIW.^[^ ^103^ ^]^ Generally, hybrid 3D printing is also appropriate for DIW. Valentine et al.^[^ ^117^ ^]^ combined DIW printing of conductive and dielectric elastomeric materials with automated pick‐and‐place of surface mount electronic components to produce a soft electronic device, which shows a great potential in wearable electronics or biomedical devices (Figure 4b).

Shear‐thinning behavior under certain shear stresses and relatively high elastic modulus are the key points for DIW. In general, the rheological behavior of common yield stress fluids can be well represented by the Herschel–Bulkley model, which follows the formula:^[^ ^118^ ^]^

(1) $$\tau =\tau_{y}+K\gamma^{n}$$

where *τ* (Pa) is the shear stress amplitude, *τ*_y_ (Pa) is the yield stress, *K* (Pa·s^n^) is the viscosity parameter, and *γ* (s^−1^) is the shear rate. The shear stress plays a critical role in tuning the microstructure of printed materials by inducing anisotropic materials, such as 1D fibers and 2D sheets. Such a microscopic orientation arrangement is capable of forming directed micro/nanoscale channels for electron, ion, and molecular transport, facilitating the development of advanced materials for high‐performance thermal conductivity, energy storage, catalysis, and water treatment devices and systems.^[^ ^119^, ^120^, ^121^ ^]^ Beyond the yielding point, the ink exhibits a shear thinning behavior and the shear tests are extremely affected by shear banding, transient effects, and shear induced fracture.^[^ ^122^ ^]^ Modulus is a key parameter to determine the self‐supporting capacity of the printable inks, and consists of the storage modulus (*G*´) and the loss modulus (*G*˝). The crossover stress (*G*´ = *G*˝) should be high enough to provide the self‐supporting capacity in the absence of stress (≈500 Pa). However, the crossover stress should also be controlled to a suitable interval (<2500 Pa), as too high of a stress could result in clogging or filter press effects for filled systems.^[^ ^123^ ^]^ Depending on the ink properties, DIW is not only capable of patterning materials through layer‐by‐layer deposition, but enables vertical printing to form array architectures (Figure 4c).^[^ ^121^, ^124^, ^125^, ^126^, ^127^ ^]^ The solidification of these inks is mainly achieved through liquid evaporation, gelation, or a certain temperature‐ or solvent‐induced phase change for direct use or postprocessing. The ink‐flow shear force renders the anisotropic structure to align parallel to the direction of the print filaments, resulting in greatly improved performance of the printed products. For example, Xia et al.^[^ ^128^ ^]^ developed a novel approach for the fabrication of short CFs reinforced SiC filaments with core–shell microstructure by DIW (Figure 4d). Contributing the high orientation of CFs in the matrix, the fracture resistance of the composite is highly enhanced, which shows great potential in the field of engineering materials. Meanwhile, DIW as a versatile printing technique also enables multimaterial 3D printing by using multiple nozzle extruders or switchable resin vat systems. Skylar‐Scott et al.^[^ ^129^ ^]^ reported the design and fabrication of voxelated soft matter via multimaterial (four‐material) multinozzle (4 × 4‐nozzle) 3D printing or a 4 × 1 two‐material multimaterial multinozzle 3D printing with high resolution of the printing process. This method substantially broadens the fabrication of voxelated materials that can be designed in complex motifs. Although FFF also enables the deposition of different thermoplastics by using multiple extruders, it is often limited by the material types, ink properties, and the relatively poor interfacial bonding. In contrast, various inks involving shape memory polymers,^[^ ^130^, ^131^ ^]^ magneto‐active soft materials,^[^ ^132^ ^]^ liquid crystal elastomers,^[^ ^133^, ^134^, ^135^ ^]^ and conductive polymers^[^ ^136^ ^]^ are successful for DIW technique. DIW is also compatible with other printing processes. For example, Peng et al.^[^ ^137^ ^]^ presented a new hybrid multi‐material 3D printing system that combines DIW with digital light processing printing, which allows for high‐speed and high‐resolution printing of multiple viscoelastic materials with complex geometry. The authors also demonstrated that a wide choice of inks and resins can be printed by this hybrid 3D printing system. Meanwhile, it provides the printed composites with tunable mechanical properties, enhanced interfacial bonding, and multifunctionality.

The meniscus‐guided DIW enables to produce diverse functional 3D architectures in micro‐ and nanometer scale length.^[^ ^110^, ^138^, ^139^, ^140^, ^141^, ^142^, ^143^, ^144^, ^145^, ^146^, ^147^ ^]^ Various materials including quantum dot,^[^ ^110^ ^]^ polymer nanowires,^[^ ^138^, ^139^, ^140^, ^144^ ^]^ metal nanoparticle,^[^ ^141^ ^]^ perovskites,^[^ ^143^, ^145^ ^]^ and metal‐organic frameworks,^[^ ^146^ ^]^ and nanodiamonds^[^ ^147^ ^]^ can be used in meniscus‐guided DIW, with higher resolution than extrusion‐based DIW. Lee et al.^[^ ^144^ ^]^ fabricated a metallic high‐aspect‐ratio atomic force microscopy probe through one‐step, on‐demand electrohydrodynamic 3D printing method, and the developed nano‐probe exhibits a better fidelity in deep trench atomic force microscopy imaging than a standard pyramidal probe (Figure 4e). The meniscus‐guided DIW is also capable of vertical 3D printing. Bae et al.^[^ ^110^ ^]^ demonstrated the vertical 3D nanoprinting of polymer nanowires embedded with carbon quantum dots. A femtoliter meniscus was used to guide the solidification of liquid inks to form vertically freestanding nanopillar structures, and the brightness is tunable by simple adjusting the height of the 3D pixels. Chen et al.^[^ ^143^ ^]^ developed a noncontact 3D nanoprinting to achieve parallel fabrication process of clustered nanowires by using a multibarrel pipet. In contrast to the extrusion‐based DIW that needs high viscosity and adequate elastic modulus for vertical 3D printing, there is no need the ink's high viscosity and elastic modulus in the meniscus‐guided DIW, a low viscosity of 1.1 mPa·s is capable of vertical 3D nanoprinting. Combined the features and advantages of the extrusion‐based and meniscus‐guided approaches, DIW is capable of producing various functional materials in micro‐ and nanometer scale.

### 3.3 Stereolithography

**Figure 5:**
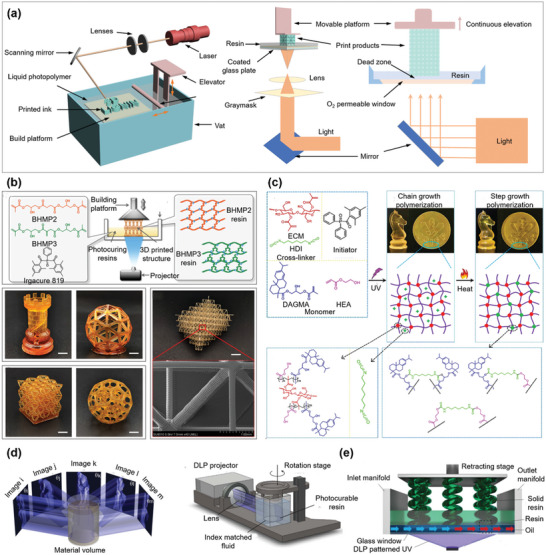
Schematic of SLA 3D printing technique and the applications of SLA printed materials. a) Schematically showing the main structure and process of a DIW printer. b) SLA printing of biobased acrylates, including the structures of acrylate monomers with di‐ or tri‐functionality (BHMP2 and BHMP3) and initiators (Irgacure 819), as well as, the cross‐linked BHMP2 and BHMP3 resins. Various 3D structures printed by BHMP2 and BHMP3 resins, respectively, and the scanning electron microscopy (SEM) image with a 200 µm resolution for printed BHMP3 resin. Reproduced with permission.^[^ ^162^ ^]^ Copyright 2020, American Chemical Society. c) Synthesis of SLA printed thermoset by two‐step polymerization, namely, the chain growth UV polymerization by rosin derived monomers (DAGMA) and 2‐hydroxyethyl acrylate (HEA) that reacted under initiator of diphenyl(2,4,6‐trimethyl‐benzoyl)phosphine oxide and cross‐linkers of methacrylated ethyl cellulose macromonomer (ECM) and hexamethylene diisocyanate (HDI), and the step growth heat polymerization to increase the crosslinking density of the printed thermoset. Reproduced with permission.^[^ ^164^ ^]^ Copyright 2020, Wiley‐VCH. d) Computed axial lithography volumetric fabrication, including the patterned illumination from different directions to a photoresponsive resin and the schematic of the computed axial lithography equipment. Reproduced with permission.^[^ ^165^ ^]^ Copyright 2019, AAAS. e) Schematically showing the rapid, large‐volume, thermally controlled 3D printing using a mobile liquid interface. Reproduced with permission.^[^ ^166^ ^]^ Copyright 2019, AAAS.

SLA is also called vat photopolymerization, which builds 3D solids in a liquid bath by selectively polymerizing and cross‐linking of a photoinitiator and a reactive monomer with an UV light.^[^ ^148^, ^149^, ^150^ ^]^ A representative SLA system is mainly composed of a bath for liquid photopolymer, a movable platform, and a laser UV source capable of emits UV light at a specific wavelength (**Figure** **5**a). The main machine setups can be divided into two types, top‐down and bottom‐up, both of which build 3D structures through a layer‐by‐layer printing sequence, and the main difference is whether the UV light used above or under the resin bath.^[^ ^148^, ^151^ ^]^ In bottom‐up SLA, UV light exposure is performed sequentially and moves in a pre‐designed path under the resin bath to solidify the lower surface of the photosensitive material. Then, the platform moves up a given distance to allow fresh, uncured resin to replenish this zone, and the 3D object is completed by repeating this process. In top‐down SLA, the UV source moves above the resin bath and the solidified upper surface moves down to replenish the fresh and uncured resin. Many commercial printers employ the configuration of bottom‐up SLA, because it requires less resin, can make 3D patterns with larger volumes, and prevents oxygen inhibition during photopolymerization, but needs a transparent bottom to allow the light to pass through. SLA printing affords the highest resolution (6–140 µm) among other 3D printing technique due to the accurate control of space and time by applying one photon or two‐photo propagating light.^[^ ^152^, ^153^ ^]^ Reports have demonstrated that nanoscale printing quality can be obtained by using two‐photon laser initiation, and the evanescent light shows unique advantages of avoiding over‐curing, thus enhancing printing quality. However, SLA printing is a time‐consuming process especially for the deposition of the new layer in the vertical direction, causing a relatively slow printing speed. Meanwhile, the SLA printer is also more expensive ($3–10 k) than FFF and DIW printers. The photopolymers used also exhibit shrinkage and brittleness that are related to the polymerization reaction.^[^ ^154^, ^155^ ^]^ The resin photo‐properties, rheological behavior, and polymerization reaction have an important effect on the process of SLA printing. The used resin must have a sufficiently low viscosity to ensure it flows smoothly into the vacated reaction zone due to the movement of the platform. Diluent and chain transfer agents are generally added to the monomer/oligomer and photoinitiators to improve the rheological properties and printability of the system.^[^ ^156^, ^157^ ^]^ The diluent agents are capable of adjusting the viscosity of the resin to endow the system with proper wetting and impregnating, and the chain transfer agents are mainly used to modify the crosslinked network.

The high resolution of SLA printing allows to fabricate various nanocomposites, such as biomaterials,^[^ ^158^ ^]^ engineering materials,^[^ ^159^ ^]^ electronic and magnetic materials,^[^ ^160^, ^161^ ^]^ etc. SLA also shows flexibility in constructing various complex patterns (Figure 5b).^[^ ^162^ ^]^ However, the presence of nanoscale composition introduces issues with printing accuracy due to the change in light scattering caused by the nanocomposition. Therefore, parameters that affect printing should be optimized to improve printing quality. First, the curing precision is closely related to the curing width (*C*_w_) and curing depth (*C*_d_) with the theoretical expression as follows:^[^ ^163^ ^]^

(2) $$C_{w}=\;F\sqrt{\ln\frac{E}{\psi E_{c}}}$$

(3) $$C_{d}=\;D_{p}\ln\frac{E}{E_{c}}$$

where *D*_p_ is the penetration depth, *E* and *E*_c_ are the exposure and the critical exposure to initiate polymerization, respectively. For example, the large refractive index difference between nanoparticles and liquid resin results in a significantly scattered laser light, thus leading to an insufficient *D*_p_, an increased *C*_w_, and an attenuated *C*_d_. The wavelength of the laser light (300–400 nm) is another important parameter, which should be carefully chosen to make it match the photoinitiator. Therefore, for a good SLA printing process, several factors, including the intensity of the laser light, rheology and wetting behavior of the resin, curing precision and composition, should be well controlled.^[^ ^148^ ^]^ In recent years, SLA has been widely used for various applications. For example, Lu et al.^[^ ^164^ ^]^ developed a robust, fluorescent shape‐memory thermoset by two‐step SLA printing, involving UV‐induced chain‐growth polymerization and heat‐induced step‐growth polymerization (Figure 5c). The printed thermoset can be used as a scaffold to fabricate flexible conductive hydrogels for smart photoelectric applications. By dynamically controlling the directions and rotation degree of photosensitive material of light, volumetric tomographic reconstruction featuring as small as 0.3 mm in engineering acrylate polymers can be achieved (Figure 5d).^[^ ^165^ ^]^ Moreover, a rapid, large‐volume, thermally controlled 3D printing using a mobile liquid interface can also be realized for SLA (Figure 5e),^[^ ^166^ ^]^ which has been successfully demonstrated on hard plastics, ceramic precursors, and elastomers.

### 3.4 Selective Laser Sintering

**Figure 6:**
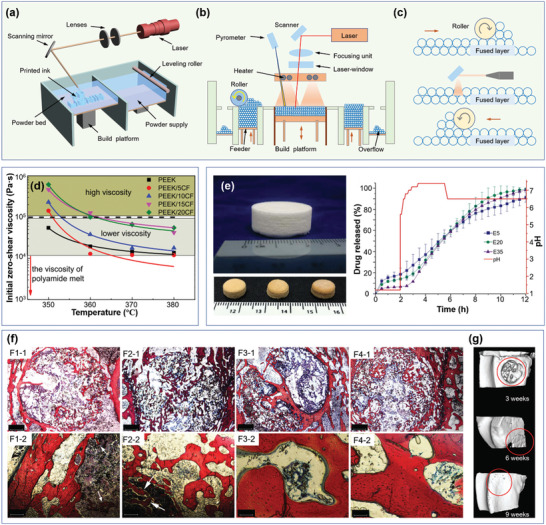
Schematic diagram of SLS 3D printing technique and the applications of SLA printed materials. a) Schematically showing the main structure of a SLS printer. b) Schematic to show the front of a SLS printer. c) Layer‐by‐layer process during SLS printing. d) The zero‐shear viscosity of polyether‐ether‐ketone and carbon fiber/polyether‐ether‐ketone composites with temperature during selective laser sintering. Reproduced with permission.^[^ ^176^ ^]^ Copyright 2018, Elsevier. e) SLS 3D printing of medicines for drug release; printed polymers with drug loadings of paracetamol (acetaminophen, 5%, 20%, and 35%) show slow release and pH‐independent release characteristics. Reproduced with permission.^[^ ^182^ ^]^ Copyright 2018, Elsevier. f) New bone formation promoted by pure poly‐*ε*‐caprolactone and 15% nano‐hydroxyapatite/poly‐*ε*‐caprolactone scaffolds constructed by SLS. g) The reconstructed images by micro‐computed tomography at 3, 6, and 9 weeks for nano‐hydroxyapatite/poly‐*ε*‐caprolactone scaffolds treated bone. Reproduced with permission.^[^ ^183^ ^]^ Copyright 2013, Dove Press Ltd.

SLS is a powder‐based additive manufacturing process that is generally used for the rapid prototyping of a complex 3D pattern, and combines the advantages of high precision and performance (**Figure** **6**a).^[^ ^167^, ^168^, ^169^ ^]^ The technique SLS was first developed by Carl Deckard,^[^ ^170^ ^]^ which was equipped with a neodymium‐doped yttrium aluminum garnet (Nd: YAG) laser and had a low power of only 100 W. Currently, the commercially available SLS mainly employs a CO_2_ laser as the heat source. The Nd: YAG laser has also been used independently or in combination with the CO_2_ laser as in the dual‐beam technique.^[^ ^167^ ^]^ The CO laser is also developed, which has an ultra‐fine spot size, with the diameter of the laser beam being approximately half that of the CO_2_ laser, permitting a higher printing precision.^[^ ^171^ ^]^ Diode or fiber lasers can also supply a comparable laser power and are much cheaper than that of CO_2_ laser, which may become mainstream in the future market.^[^ ^172^ ^]^ In addition, the sintering machine currently provides a wide tunable power ranging from 50 to 200 W and is appropriate for a wide range of materials. The SLS apparatus mainly consists of a control computer, a supply platform, a laser, galvo mirrors, a powder reservoir platform or hopper, a mechanical roller, and a material vat.^[^ ^173^ ^]^ The laser is responsible for the sintering process and the galvo mirrors direct the laser beam to a correct position. During printing, the surface of the powders is exposed to the laser and selectively sintered and fused to form a desired shape for each 2D layer (Figure 6b). In this process, when heat energy from a laser is absorbed by the powder bed, the powders bind through a complicated phase transition process or mechanism, including viscous flow binding, curvature effect, wetting, solid‐state and liquid‐phase sintering, and full melting. Once the sintering of a powder layer is finished, the fresh power is transferred and flattened by a mechanical roller to the supply platform for recoating the printed layer (Figure 6c), and the unsintered power will be recovered by a material vat. The lasers inject the thermo‐energy to the powder for sintering and fusion, with an operation principle similar to the FFF process, yet the SLS printing shows a higher resolution (≈60 µm) and does not require the pre‐processing of its starting material, which makes it one of the most versatile technologies.

The structural properties and performance of the 3D object are directly dominated by the processing parameters, including temperature, laser power, laser scanning speed, scan spacing, spot size, powder size distribution, layer thickness, diversity of feedstock, binder volume fraction, etc.^[^ ^174^ ^]^ Powder bed temperature is an important parameter that controls and promotes the sintering process, which is mainly dependent on the properties of polymers. For thermoplastic amorphous polymers, the temperature of the powder is generally set to above the glass‐transition temperature (*T*_g_) to enable their consolidation. For crystalline polymers, the temperature is commonly slightly lower than the melting temperature (*T*_m_) but higher than the crystallization temperature (*T*_c_) to melt the polymer, meanwhile, to retard crystallization. In the case of semi‐crystalline polymers and polymer mixtures, the temperature is usually set near the *T*_g_, which can be calculated by the following equation:

(4) $$1/T_{g}=W_{1}/{T^{\prime }}_{g}+W_{2}/{T^{\prime \prime }}_{g}$$

where the *W*_1_ and *W*_2_ are the weight fractions of each polymer, and the *T*´_g_ and *T*˝_g_ refer to the *T*_g_ of each pure polymer, respectively.^[^ ^175^ ^]^ Yan et al.^[^ ^176^ ^]^ analyzed the high temperature rheological behavior and sintering kinetics of CF/polyether‐ether‐ketone (CF/PEEK) composites during SLS printing, and found that the zero‐shear viscosity of PEEK and its composites decreased with temperature, and the composites displayed a high coalescence rate at low viscosity (Figure 6d). SLS is an energy‐consuming process, thus, the wavelength of the laser beam plays an essential role in the sintering process of polymers. To adapt to the absorptance of the powders and to yield an appropriate powder bed temperature, the energy transmittance of the laser beam is often adjusted by modulating the fine‐tunable laser power and scanning speed. The laser scanning speed can highly influence the laser energy density on the powder surface, and the lower speed is generally used to allow a higher energy transmission, higher sintering degree, and denser objects.^[^ ^177^ ^]^ The scan spacing should be set based on the diameter and energy density of the laser beam, and a scan spacing that is too large often leads to incomplete sintering. As a result, the yielded objects display a low mechanical property. Likewise, a scan spacing that is too small may induce thermal deformations, therefore, although decreasing the scan spacing is best for fabricating a thin and intricate structure, the decrease should not exceed the limit. The powder size distribution also plays a significant role in the sintering process. The big particles will result in low mechanical strength, while too small particles often cause a poor flow property due to the high electrostatic force. Leong et al.^[^ ^178^ ^]^ pointed out that spherical particles with sizes ranging from 58 to 180 µm are desired and appropriate for SLS printing. To ensure accuracy, the layer thickness should be slightly higher than the average particle size and typically ranges from 0.07 to 0.5 mm to obtain a high resolution.^[^ ^179^ ^]^ Due to the complexity of SLS printing, many other parameters affect the structure and performance of the object, such as the dwell time^[^ ^180^ ^]^ and the building orientation (e.g., horizontal, vertical, or diagonal).^[^ ^181^ ^]^ Therefore, these parameters should be comprehensively considered for a proper sintering step to obtain a desired material. SLS printing is mainly applied for production of medicines, such as tablets for slow drug release and biological tissue repair.^[^ ^167^ ^]^ Fina et al.^[^ ^182^ ^]^ fabricate tablets with cylindrical, gyroid lattice, and bilayer structures. The gyroid lattice structure shows a customizable release characteristic for paracetamol (Figure 6e). Xia et al.^[^ ^183^ ^]^ constructed a nano‐hydroxyapatite/poly‐*ε*‐caprolactone (nano‐HA/PCL) scaffold by SLS printing for bone tissue engineering applications, and the nano‐HA/PCL exhibited better biocompatibility and enhanced efficiency of new bone formation than PCL (Figure 6f,g). These results indicate that SLS printing shows large potential for use in medicine.

## 4 Structure‐Rheology Relationship of Lignin in 3D Printing

The structure and performance of printed materials mainly depend on the 3D printers, the molecular structure, and morphology of feedstocks. In which, the structure‐rheology relationship has a decisive effect on the printability, the performance, and application of 3D scaffolds. The differences in printability of a polymer arise primarily due to the rheological characteristics both in the printing state and in their solid form. Further, the addition of fillers or additives in the polymer also changes the materials’ characteristics and their printability. Therefore, summarizing the structure‐rheology relationship of lignin will be helpful for material design and smooth printing of lignin‐based composites. Lignin, as a biopolymer that features *T*_g_ and powder morphology, has been mainly employed in date on extrusion‐based 3D printing (FFF and DIW) and SLS. In addition, lignin is also used in SLA of vat‐photopolymerization, yet it mainly relies on photoactive and low viscosity resins that are generally composed of acrylic‐ or epoxy‐ based monomers. In this part, the relationship of lignin structure with melt rheology in FFF, ink rheology in DIW, powder rheology in SLS, and rheology in other 3D printing technique (mainly SLA) is critically discussed, which aims to help understand the structure‐rheology relationship of lignin and the effect of lignin on the properties and performance of the printed composites.

### 4.1 Structure‐Melt Rheology Relationship in Fused Filament Fabrication

Melting lignin to a low but sufficiently viscosity fluid is a critical process for FFF printability, where the thermal stability of lignin plays an important role in the continuous printing of the filaments. Although a reduced viscosity with increased temperature is often observed for common polymers such as PLA^[^ ^184^ ^]^ and polyether ether ketone,^[^ ^185^ ^]^ this strategy is not applicable for technical lignins due to their poor thermal stability. Technical lignins from paper or biorefinery mills often display a wide polydispersity in molecular weight,^[^ ^186^ ^]^ resulting in the printing temperature being difficult to control. At low temperature, the large molecular weight of lignin is hard to melt, while at high temperature the low molar mass lignin degrades to form a rigid char, both leading to increased resistance to flow or deformation. Organosolv hardwood lignin with abundant linear sinapyl alcohol units has been reported to exhibit good melt stability.^[^ ^187^, ^188^ ^]^ The organosolv hardwood lignin is dominant in *β*‐O‐4 linkages and contains long aliphatic C*_*α*_*—C*_*β*_*—C*_*γ*_*—OH chains, meanwhile, presents a less rigid aliphatic structure, which endows the lignin segments with good flexibility.^[^ ^189^ ^]^ However, for large molecular weight fraction of hardwood lignin, the considerable steric hindrance and large free molecule volume caused by S units and C_*α*_‐carbonylated S units leads to an increased molecular mobility, thus reducing the *T*_g_ and melt viscosity.^[^ ^190^, ^191^ ^]^ The abundant methoxy, aliphatic ether, and oxygenated aromatic carbon groups in hardwood lignin are reported to provide a high degree of freedom for rotating and molecules bending.^[^ ^190^, ^191^ ^]^ Similarly, with abundant condensed G units, kraft softwood lignin does not flow well at high temperatures unless the extracted oligomeric fraction (≈1000 Da) is used.^[^ ^192^, ^193^, ^194^ ^]^ The poor thermal stability of kraft softwood lignin is mainly caused by the presence of a substantial amount of stiff segments that are composed of biphenyl and biphenyl ethers based on G and H units, leading to a higher *T*_g_ than hardwood lignin.^[^ ^189^, ^195^ ^]^ Moreover, compared with hardwood lignin, softwood lignin also presents higher amounts of rigid aromatic methane carbons and C_Ar_—H, which make a huge difference in rheology behavior. Nguyen et al.^[^ ^189^ ^]^ compared the rheology behavior between softwood and hardwood lignin at two temperatures (170 and 190 °C), and the results revealed that at ≈1% strain softwood lignin shows a shear‐thinning behavior, whereas hardwood lignin has a much longer elastic response with a strain amplitude over 10%. Softwood lignin shows much higher shear stress in the linear region and presents a stronger solid‐like behavior than hardwood lignin. The storage (*G*′) and loss (*G*′′) modulus are also important parameters to evaluate the viscosity and self‐supporting capacity. The frequency‐dependent *G*′ of both softwood and hardwood lignins displays a similar trend, but the *G*′ value of softwood lignin is several orders of magnitude larger than that of hardwood lignin, which indicates that the softwood lignin has much higher complex viscosity. The high complex viscosity is not suitable for melt processing and easily causes high resistance to flow. In summary, oligomeric lignins with narrow polydispersity and viscosity of <1000 Pa·s are available for consistent extrusion to a fiber or filament. At the molecular level, lignin with abundant aliphatic ether, *β*‐O‐4 linkages, oxygenated aromatic bonds offers a good flow characteristics and printability. However, a very good flow behavior at processing temperatures does not mean that these oligomeric lignins are better candidates for 3D printing applications. The filaments and printed structures by these neat phenolic oligomers are generally very brittle and cannot form a self‐supporting structure alone. Therefore, the extrudability of the oligomer lignin filaments does not necessarily assure a good printability in FFF and the direct use of pristine lignin for 3D printing remains limited.

**Figure 7:**
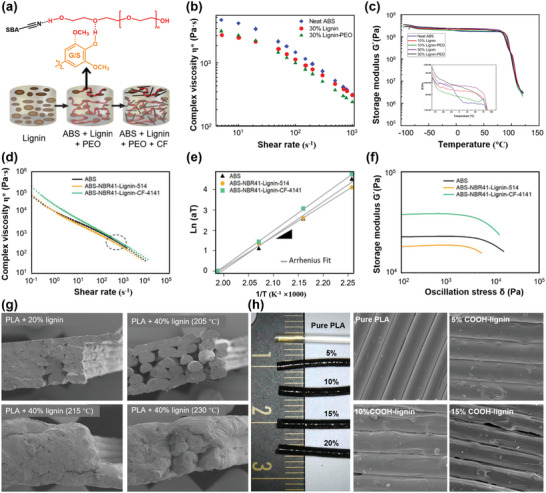
Rheological behavior of lignin‐based blends for FFF. a) Schematic of poly(ethylene oxide)‐assisted macromolecular self‐assembly of lignin in an ABS matrix. b) Change in the viscosity of neat ABS, lignin/ABS, and lignin/ABS/PEO blends with shear rate at 200 °C. c) Relationship between *G*′ and temperature for ABS, lignin/ABS, and lignin/ABS/PEO blends at 1 Hz frequency. Reproduced with permission.^[^ ^192^ ^]^ d) Shear rate dependent viscosity of ABS, ABS‐NBR41‐lignin‐514, and ABS‐NBR41‐lignin‐CF‐4141 at 230 °C. e) The linear relation between the shift factor (aT) and the inverse temperature (1/T). f) *G*′ as a function of oscillation stress at 230 °C. Reproduced with permission.^[^ ^199^ ^]^ Copyright 2018, Elsevier. g) SEM images of printed soda lignin/PLA belnds and the fracture surface with different lignin addition and temperature. Reproduced with permission.^[^ ^205^ ^]^ Copyright 2018, MDPI. h) Digital and SEM images of printed carboxylated lignin/PLA blends with different lignin content. Reproduced with permission.^[^ ^206^ ^]^ Copyright 2021, MDPI.

In most cases, lignin is often blended with a printable polymer or copolymerized with a soft segment to increase the toughness of the extruded polymers. The polymer matrix or soft segment for FFF mainly includes PLA,^[^ ^196^ ^]^ ABS,^[^ ^192^ ^]^ graphene,^[^ ^197^ ^]^ nylon12,^[^ ^189^ ^]^ polyhydroxybutyrate,^[^ ^198^ ^]^ methacrylate,^[^ ^190^ ^]^ polyethylene oxide (PEO),^[^ ^43^ ^]^ CF,^[^ ^189^ ^]^ etc. These polymers or soft segments can be used alone or as a mixture to improve the melt rheology and the inter‐layer adhesion, and make the composition strong and tough. For example, with good FFF compatibility, mechanical performance, and melt properties, ABS is the commonly used polymer for blending with lignin to improve the printability. Compared with neat ABS, the viscosity of ABS/lignin generally decreases with lignin loadings at low shear rate regions (1–500 s^−1^) and tends to be similar at high shear rate regions (500–1000 s^−1^), exhibiting a power law shear thinning behavior.^[^ ^192^ ^]^ However, the poor bonding energy between the printed layers and the excessive brittleness of the resulting composites particularly at high lignin loadings limit the printed materials’ applications. Therefore, the incorporation of PEO, cellulose nanocrystals (CNC), acrylonitrile butadiene rubber (NBR) or graphene is often conducted to increase the adhesion between lignin and ABS. Akato et al.^[^ ^192^ ^]^ studied the effects of PEO on the melt rheology of ABS/lignin, and the results revealed that the presence of PEO lowers the viscosity of ABS/lignin and improves the melt‐processability of the blends without reducing the *G*′ value (**Figure** **7**a–c). Nguyen et al.^[^ ^199^ ^]^ integrated lignin with NBR and ABS for FFF printing. The usage of NBR also greatly improves the melt‐processability of the blends and enhances the toughness of the composites. The authors also further evaluated the melt rheology and 3D‐printing characteristics of the blends (50 wt% ABS, 10 wt% NBR, and 40 wt% lignin vs 40 wt% ABS, 10 wt% NBR, 40 wt% lignin, and 10 wt% carbon fiber) using the Cox‐Merz rule and found that the former shows the lowest melt viscosity in the range of 10^2^–10^4^ s^−1^ (Figure 7d–f). Although the addition of CF increases the melt viscosity, the value is not higher than that of neat ABS and the blends without CF at a high shear rate. For 3D printing, good shear thinning behavior, low melt viscosity, and zero‐shear viscosity in the terminal region, are desirable to have good printability and maintain the 3D shape after deposition. High yield stress is important to retain the dimensions of the 3D scaffolds. The yield stress of both blends (2 × 10^3^ and 3 × 10^3^ Pa, respectively) can meet the requirements of 3D printing applications. As a result, the printed composites by using these polymers exhibit good printability and excellent mechanical performance.

Some polymers used alone can also improve the printability of lignin/polymer blends for 3D printing applications, such as nylon 12 and PLA. Nguyen et al.^[^ ^189^ ^]^ comprehensively studied the effects of nylon 12 on the melt rheology and printability of hardwood lignin. The hardwood lignin particles cause a low melt viscosity to nylon 12 and show a significant reinforcement at room temperature, yet result in plasticization in the melt. At 190 °C, the hardwood lignin particles have a low melt viscosity of 100 Pa∙s in the frequency range of 10–100 rad s^−1^ and behave like a lubricant phase, which can highly mobilize the nylon macromolecules. In addition, the melt viscosity of the nylon 12 drops rapidly as the lignin content increases from 40 to 60 wt%. For example, the complex viscosities of the lignin/nylon 12 blend drop sharply from 150 to 32 Pa∙s once the lignin content increases from 40 to 60 wt% at 230 °C and an angular frequency of 100 rad s^−1^, which provides good flow characteristics of required viscosity and shear rate windows for 3D printing. During the 3D printing process, the molten polymer blends are extruded to form a filament and deposited layer‐by‐layer into a 3D scaffold. The significantly reduced shear rate on the newly printed layer requires a relatively high *G*′ and yield stress for the extruded filament to maintain the shape of the printed 3D object. Generally, materials with viscosities between 20 and 280 Pa∙s, moduli ranging from 10^3^ to 10^5^ Pa, and yield stresses from 150 to 10^3^ Pa are necessary for good printability.^[^ ^90^, ^109^, ^200^, ^201^, ^202^, ^203^ ^]^ For the lignin/nylon 12 blend, both the *G*′ and yield stress are controllable, and can be highly comparable to the required values to retain the structural stability after deposition. The addition of CF has no negative effects on printability and can improve the mechanical properties of the composites, but the melt viscosity of the blend will increase.

In addition to the polymer nylon 12, it has been reported that the PLA matrix also shows excellent compatibility with organosolv hardwood lignin, and 15 wt% lignin loading endows the blend with good printability by FFF. In contrast, with an equivalent softwood kraft lignin, the lignin/PLA blend shows poor printability, which is primarily attributed to the degradation of PLA caused by the residual soda in lignin.^[^ ^204^ ^]^ However, Tanase‐Opedal et al.^[^ ^205^ ^]^ found that the PLA with lignin loading of 40 wt% can also exhibit very good printability (Figure 7g), despite different structural properties of lignin, where the printing temperature may dominate the printability. Chemical modification of lignin is another effective strategy to improve the printability of lignin/polymer blends. For example, the carboxylated OSL can effectively improve the PLA interfacial adhesion between the deposited layers through hydrogen bonding. A carboxylated lignin content of 10 wt% is considered to be the most cost‐effective for FFF printing and a content 20 wt% would cause a remarkable decrease in the tensile modulus (Figure 7h).^[^ ^206^ ^]^

### 4.2 Structure‐Ink Rheology Relationship in Direct Ink Writing

**Figure 8:**
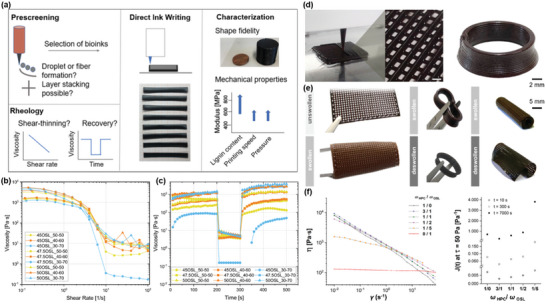
Rheological behavior of lignins and lignin‐based blends for DIW. a) Schematic to show the optimization of the formulations and DIW parameters for fully bio‐based OSL/HPC inks. b) Viscosity as a function of shear rate for different formulations. c) Recovery tests at high shear rate of 895 s^−1^ (200–300 s) and low shear rate of 0.01 s^−1^ (0–200 s and 300–500 s) for OSL/HPC inks. Reproduced with permission.^[^ ^208^ ^]^ Copyright 2020, American Chemical Society. d) DIW of HPC/organosolv lignin blend solutions. e) Swelling behavior of printed composites cross‐linked with citric acid and dimerized fatty acid. f) Viscosity as a function of shear rate and the changes in creep compliance with the HPC/OSL ratio at 25 °C. Reproduced with permission.^[^ ^212^ ^]^ Copyright 2020, American Chemical Society.

DIW is an extrusion‐based 3D printing technique that can process the printable polymer ink at room temperature by using shearing force to rapidly fabricate various 3D patterns. Different from FFF, there is no need to heat lignin or lignin/polymer blends above a melting or softening point for DIW. Instead, the main challenge of DIW is to design a viscoelastic ink that can easily flow through the nozzle and is capable of forming a free‐standing structure. Therefore, colloidal gels or organic inks with good shear‐thinning behavior and low zero shear rate compliance are particularly suited for DIW.^[^ ^103^ ^]^ Unlike the main constituent of cellulose in lignocellulose, lignin shows unfavorable rheological behavior by directly mixing with water or organic solvents, which prevents the smooth printing of lignin and tends to clog the printing nozzle.^[^ ^207^ ^]^ At present, the implementation of DIW on lignin always relies on polymer blending to form colloidal gels or pastes, such as cellulose nanofibers (CNFs) and their derivatives,^[^ ^208^, ^209^, ^210^, ^211^, ^212^ ^]^ carbon‐based polymers,^[^ ^213^ ^]^ and viscoelastic polymers.^[^ ^207^, ^214^ ^]^ For example, Ebers and Laborie^[^ ^208^ ^]^ optimized the formulations and DIW parameters for fully bio‐based OSL/hydroxypropyl cellulose (OSL/HPC) inks (**Figure** **8**a). The developed bio‐based OSL/HPC ink shows obvious shear‐thinning and the viscosity increases with OSL/HPC ratios (w/w, Figure 8b). Recovery tests indicate that the formulations with an OSL/HPC of 30/70 (w/w) exhibit the lowest viscosity recovery, while that of 50/50 (w/w) display a rapid viscosity recovery (Figure 8c). Lignin provides stability and solid‐like properties and HPC confers the needed shear‐thinning to the blends at rest, resulting in a desirable viscoelastic behavior for DIW.

With strong shear‐thinning properties and low zero shear rate compliance, CNF hydrogels have been widely used as a material for DIW, and are also compatible with lignin. Zhang et al.^[^ ^211^ ^]^ used spherical colloidal lignin particles (CLPs) to prepare Ca^2+^‐crosslinked CNF‐alginate‐CLP nanocomposites by DIW. The resulting CNF‐alginate‐CLPs hydrogels demonstrate good shear‐thinning behavior, in accordance with that of the CNF‐alginate hydrogel (without lignin). The viscosity of CNF‐alginate‐CLPs hydrogels increases with CLP concentration ranging from 5 to 25 wt% but decreases at 1.0 wt% (based on CNF). In general, the viscosity of CNF‐based hydrogels with contents of solid additive lower than 4% mainly depends on the CNF concentration, and the increased zero shear rate viscosity by adding lignin has been demonstrated to improve the printing resolution and shape fidelity of printed scaffolds. CNF‐alginate‐CLPs hydrogels display a gel‐like nature with the obtained tan(*δ*) value (*G*′′/*G*′) less than 1.0, suggesting the high stability of 3D objects. However, it is worth noting that the lignin content in final ink only accounts for 0.5 wt%, which limits its application on a large scale. Gleuwitz et al.^[^ ^212^ ^]^ further present a lignin‐containing liquid crystalline network material fabricated by DIW (Figure 8d). The liquid crystalline ink was constructed through HPC blending with 25 wt% beech OSL in an acetic acid solution. By esterification with citric acid and dimerized fatty acid, the obtained printed structure exhibits good water insolubility and anisotropic swelling (Figure 8e). The same group further optimized the formulations and DIW parameters for the HPC/lignin inks and increased lignin content up to 50 wt% after a series of rheological analyses. Interestingly, neither HPC nor OSL used alone can be printed by DIW, but the blend of these two bio‐based polymers shows good shear‐thinning properties, improved printability, and high shape fidelity using the lignin stabilizing effect. The stabilizing effect of lignin mainly stems from its phenolic hydroxyl groups, low molecular weight, and polydispersity. A positive deviation is observed from the law of the creep compliance (*J*) of blend solutions (*ω*_p_ = 0.6) as a function of the HPC/OSL ratio (at 25 °C, Figure 8f). The low creep compliance for the 3/1 blend solution (25 wt% lignin) is favorable to the post‐processing stability of the printed structure. However, tedious time to prepare such inks is needed (over 7 days), and an instantaneous crosslinking or solidification remains desirable to enlarge shape freedom and throughput. The DIW of such inks is appropriate for OSL with low molecular weight and polydispersity, and a universal polymer blending is highly desirable to endow lignin with good printability.

**Figure 9:**
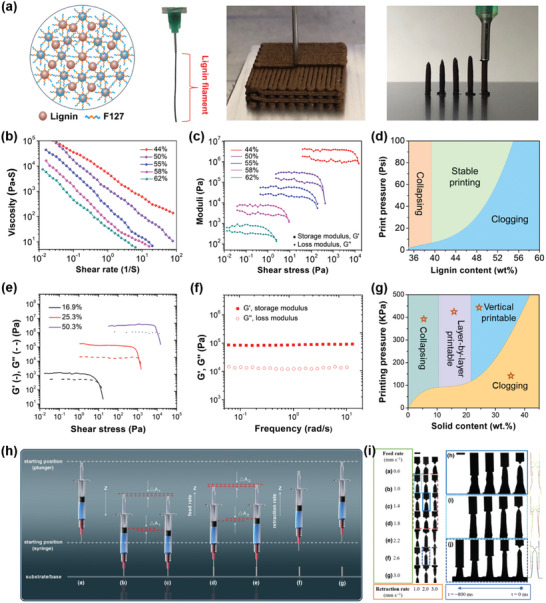
Rheological behavior and printing processes of lignins and lignin‐based blends for DIW. a) Schematic diagram of the lignin/F127 blend and digital images of its printability. b) Plot of apparent viscosity and c) *G*′/*G*′′ as a function of shear rate for lignin/F127/water inks. d) Mapping of the printability of lignin/F127/water inks. Reproduced with permission.^[^ ^207^ ^]^ Copyright 2020, Wiley‐VCH. e) *G*′/*G*′′ as a function of shear stress for the inks with different g‐C_3_N_4_/CNT/lignin loadings. f) Dynamic modulus as a function of frequency for the ink with g‐C_3_N_4_/CNT/lignin loading of 25.3 wt%. g) Mapping of the printability of g‐C_3_N_4_/CNT/lignin inks. Reproduced with permission.^[^ ^214^ ^]^ Copyright 2021, Wiley‐VCH. h) Printing steps of a single pillar in vertical printing. i) Separating moment and dynamic formation of a pillar during vertical 3D printing under different feed and retraction rates, scale size 250 mm. Reproduced with permission.^[^ ^216^ ^]^ Copyright 2020, Elsevier.

Jiang et al. developed a low‐cost and universal DIW to construct lignin‐based 3D scaffolds.^[^ ^207^ ^]^ The printable ink is prepared by direct mixing lignin with Pluronic F127 (polyoxyethylene‐polyoxypropylene‐polyoxyethylene triblock copolymer) aqueous solution (**Figure** **9**a). A series of rheology analyses were also conducted to quantitatively understand the printability of the lignin/F127 inks. The results showed that lignin/F127 inks display good viscoelastic behavior with a large shear yield strength of *G*′ and a pronounced decrease in viscosity (Figure 9b). The modulus and viscosity of the lignin/F127 inks can be easily adjusted by changing the lignin content from 38 to 56 wt%, and the yield stress as a function of shear stress shows a good linear relationship (Figure 9c). The well‐controlled rheological properties indicate that the lignin/F127 inks exhibit good flow characteristics and printability. Within the concentration range of 38–56 wt%, lignin/F127 inks are highly adjustable from soft to rigid to printed 3D objects with good self‐supporting capacity and strong interfacial adhesion between adjacent layers (Figure 9d). It has also been demonstrated that various lignins such as alkali and kraft lignins from hardwood and softwood exhibit excellent printability, which reveals this developed DIW strategy by using F127 as a soft agent is a universal way to build diverse sophisticated patterns.

Lignin/F127 inks can be vertically printed (Figure 9a). The structures or groups of biphenyls, biphenyl ethers, and aromatic methane carbons provide rigid and stiff properties to lignin, which endows the vertically extruded ink filament with outstanding self‐supporting capacity. There is no need the ink's high viscosity and elastic modulus for vertical 3D printing in the meniscus‐guided DIW, the extrusion‐based DIW, primarily depends on the ink's high viscosity and elastic modulus, generally, with an apparent viscosity >10^3^ Pa·s at a shear rate of ≈10^−1^ 1 s^−1^ and elastic modulus over 10^4^ Pa.^[^ ^202^, ^215^ ^]^ It is worth noting that the lignin content in the 3D scaffolds can be up to 83.3% after water evaporation, which is much superior to other printed lignin‐based 3D objects. Vertical printing by this method is also compatible with multiple materials. For example, Jiang et al. developed a mixed ink containing graphitic carbon nitride (g‐C_3_N_4_), carbon nanotubes (CNTs), and lignin with the ink rheology adjusted by an F127 aqueous solution.^[^ ^214^ ^]^ The apparent viscosity of the inks as a function of shear rate on a logarithmic scale displays a pronounced and nearly linear decrease with the shear rate increasing from ≈1 to 10^2^, a typical feature of non‐Newtonian fluids. Both *G*′ and yield stress increase with the concentration of the inks, and *G*′ is approximately tenfold higher than G″ within the plateau region, indicating that the inks are stiff with a solid‐like response (Figure 9e,f). Despite the excellent printability, the inks that enable vertical 3D printing only exhibit a narrow window (Figure 9g), and the ink with 25.3 wt% has been demonstrated to be vertically 3D printed meanwhile shows a good physical dispersion stability without sedimentation. Nan et al.^[^ ^216^ ^]^ provided multiple examples to investigate the process of vertical 3D printing in detail (Figure 9h). Vertical 3D printing starts with the nozzle of syringe moving downward gradually until it reaches the substrate. Subsequently, under an appropriate feed rate, the ink is extruded and deposited by the downward movement of the piston to form a pillar with as‐designed height. Then, the feed rate changes to a retraction rate, and at this moment the nozzle starts to separate from the pillar and the ink flow stops immediately. Finally, the syringe returns to the starting position by following the procedure for the next pillar deposition, thus forming an as‐designed array architecture. In an ideal scenario, the printable ink is an incompressible fluid, and the volume change in the syringe is equal to that of the extruded ink (Δ*A*_1_ = Δ*A*_2_). However, the energy dissipation is inevitable due to the friction among the ink, inner wall of the syringe, and the piston, leading to Δ*A*_1_ larger than Δ*A*_2_, which is important to maintain the printing reproducibility. Both feed rate and retraction rate are important parameters affecting the morphology of pillars (Figure 9i). At a low feed rate (0.6 mm s^−1^), a jaggy aspect generally appears on the surface, and the retraction rate has no obvious effect on the shape of the pillars. At a high feed rate (1.8 mm s^−1^), the uneven surface disappears and the tip of the pillar presents a sharp and regular contour, irrespective of the retraction rate. But further increasing the feed rate (2.2 mm s^−1^) makes the printing process unstable and the point of separation is not at the tip of the nozzle but anywhere in the pillar. The height of the pillars can hardly be controlled. Regardless the feed rate smaller, equal to, or larger than the retraction rate pillars are subjected to plastic elongation. With increasing time, the outer contour and the diameter of the printed pillar are becoming gradually narrower until the nozzle separates from the tip of the as‐printed pillar. A good combination of feed rate with retraction rate will result in a reproducible printing process and endow the printed arrays with good quality. For successful vertical printing on a glass substrate by using a nozzle diameter of 250 µm, the feed rates from 1.0 to 1.8 mm s^−1^ and retraction rates ranging from 1.0 to 3.0 mm s^−1^ are suggested by the authors.

**Figure 10:**
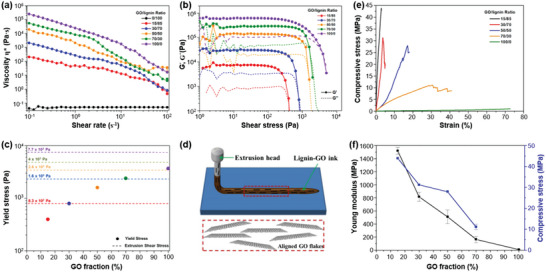
Effects of water‐soluble lignin on the rheological behavior and printing processes of lignins and lignin‐based blends. a) Viscosity of lignin/GO inks with their shear rate. b) Changes in *G*′ and *G*′′ with shear stress. c) The yield stress of lignin/GO inks with different GO fractions. d) Schematic of the alignment of GO flakes by the ink‐flow shear force during extrusion. e) Compressive stress of the carbonized 3D printed GO/lignin composites. f) Changes in Young's modulus and compressive stress with GO fraction. Reproduced with permission.^[^ ^213^ ^]^ Copyright 2020, Elsevier.

Different from the above‐mentioned results by using water‐insoluble lignin, the ink obtained by the combination of polymers with water‐soluble lignin exhibits distinctive rheological behavior. One case in point is the DIW of water‐soluble alkali lignin/graphene oxide (GO) ink demonstrated by Roman et al.^[^ ^213^ ^]^ The results of rheological analysis indicate that the viscosity of lignin/GO inks instead decreases with the amount of lignin, and the pure lignin solutions exhibit the properties of Newtonian fluids (**Figure** **10**a). The rheological behavior of lignin/GO inks mainly depends on the GO concentration, and a high GO concentration affords printability.^[^ ^217^, ^218^ ^]^ At low shear stress, the much larger *G*′ than *G*′′ for lignin/GO inks enables the formation of a stable structure after the extrusion process (Figure 10b). In addition, lignin was demonstrated to enhance the structural stability of the shear‐induced transient band texture, and enables the construction of highly oriented filaments via shear casting.^[^ ^219^ ^]^ In this work, the yield stress increases with GO content within the experiment, and the pure GO shows the highest yield stress. Generally, inks extruded with shear stress greater than the yield stress undergo a liquid‐type shear flow (Figure 10c). Under such conditions, fibers or 2D laminar structures such as cellulose,^[^ ^120^, ^220^ ^]^ GO,^[^ ^119^ ^]^ boron nitride,^[^ ^121^ ^]^ and g‐C_3_N_4_^[^ ^214^ ^]^ tend to align with the plane parallel to the filament direction. For lignin/GO inks, the extrusion shear stress is always higher than the corresponding yield stress, it means that the GO flakes are aligned in the filament direction (Figure 10d). As a result, the carbonized lignin/GO composites exhibit enhanced rigidity and mechanical strength (Figure 10e,f).

In extrusion‐based DIW, the shrinkage of the printed structure by solvent evaporation or post‐processing is a very important parameter affecting the fidelity of 3D printed lignin‐based materials, which may even cause the collapse of the structure. Generally, anisotropic shrinkage is expected as a result of molecular orientation^[^ ^212^ ^]^ and physical network can significantly reduce the volumetric shrinkage.^[^ ^221^ ^]^ Due to the high cross‐linked and rigid molecular structure of lignin, the addition of lignin often endows the 3D printed lignin‐based materials with high shape stability. For example, lignosulfonate is demonstrated that can significantly prevent the shrinkage of printed lignin/microfibrillated cellulose composites.^[^ ^222^ ^]^ In addition, formulation more drastically affects the shape fidelity than printing parameters, for example, Ebers and Laborie^[^ ^208^ ^]^ found that shape fidelity in thickness and width is more drastically affected by the HPC/OSL ratio than the solid content of the precursor lignin solution. Post‐processing will also cause the shrinkage of the printed lignin‐based materials. Roman et al.^[^ ^213^ ^]^ found that the printed structure tends to shrink by 5–10% after carbonization, which results from the elimination of oxygen and hydrogen during thermal reduction. For successful 3D printing of lignin‐based inks, the rheological behavior, process parameters, and shrinkage features should be comprehensively considered.

### 4.3 Structure‐Rheology Relationship in Selective Laser Sintering and Stereolithography

**Figure 11:**
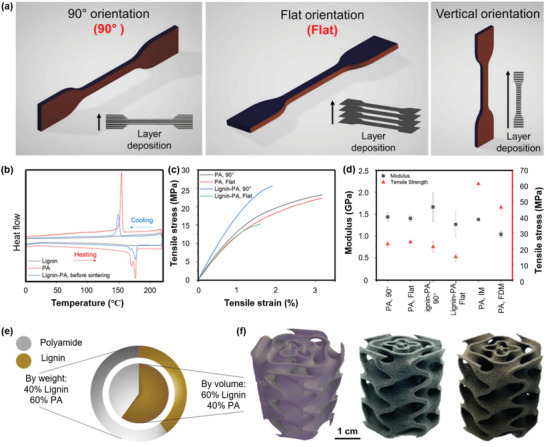
SLA printing of lignin‐based composites and their mechanical strength. a) 3D‐printedlignin/PA blends using different layer deposition directions. b) Differential scanning calorimetry of lignin, PA, and lignin/PA (60/40, v/v) before sintering. c) Tensile strain–stress behavior, and d) comparison of the mechanical strength of lignin/PA with that obtained from neat PA using SLS, FFF, and injection molding. e) Lignin loading for SLS printing. f) 3D lattice structures fabricated by SLS with PA and lignin/PA. Reproduced with permission.^[^ ^223^ ^]^ Copyright 2021, American Chemical Society.

3D printing of lignin mainly adopts FFF and DIW techniques, and few reports are focused on the SLS and SLA techniques. To the best of our knowledge, SLS printing of lignin is presently only reported by Rojas's group,^[^ ^223^ ^]^ the authors used partially replaced polyamide (PA) powder by alkali lignin as a starting material to fabricate a 3D lattice structure with flat and flipped (90°) via SLS method (**Figure** **11**a). Thermal properties have an important influence on SLS printing,^[^ ^175^ ^]^ in which the temperature of powder is usually set near to the *T*_g_ but needs to be slightly below the melting temperature (*T*_m_) to avoid polymer melting, fusion, or degradation (Figure 11b). As an amorphous polymer, it is difficult to confirm the *T*_g_ value of lignin and only a wide range can be obtained (80–180 °C), depending on lignin properties.^[^ ^224^, ^225^, ^226^, ^227^ ^]^ The authors demonstrated that the blended lignin/PA powder with 40/60 (wt%) can be successfully printed by SLS at 175 °C. The thermal properties of lignin/PA were also evaluated and the results showed that the pure PA and lignin/PA blend exhibit a fairly similar *T*_m_, yet the crystallization temperature (*T*_c_) decreased slightly after mixing with lignin. As a result, the mechanical strength of the printed structures highly depends on processing orientation (Figure 11c,d), and an object with complex geometry can be printed by a scalable SLS process, displaying a high resolution (Figure 11e,f). This low‐cost, bio‐based SLS printing strategy opens a new avenue to prepare lignin/PA composites with high lignin content, which is expected to promote wide applications in the future.

**Figure 12:**
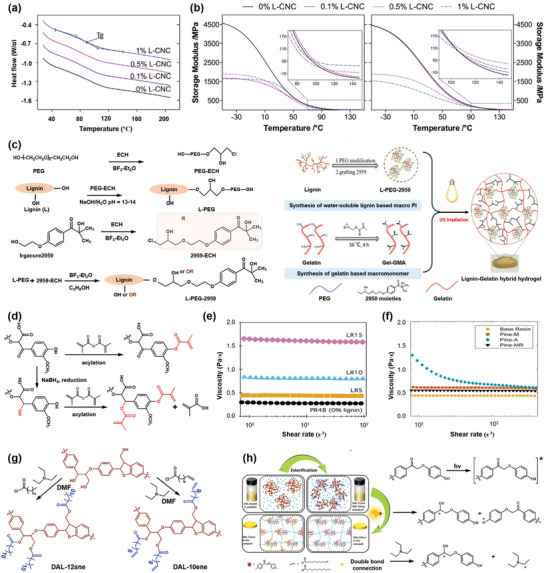
SLS printing of lignin‐based composites and their rheological behavior. a) Differential scanning calorimetry of SLA printed lignin containing CNC/methacrylate nanocomposites. b) *G*′ as a function of temperature of SLA printed lignin containing CNC/methacrylate nanocomposites before (left) and after (right) postcure. Reproduced with permission.^[^ ^229^ ^]^ Copyright 2017, Elsevier. c) Synthetic route of lignin‐PEG‐2959 and the fabrication procedure of lignin‐gelatin hybrid hydrogels. Reproduced with permission.^[^ ^232^ ^]^ Copyright 2019, American Chemical Society. d) Reduction and/or acylation of softwood lignin.^[^ ^233^, ^234^ ^]^ e) Plot of viscosity versus shear rate of acylated lignin resins. Reproduced with permission.^[^ ^233^ ^]^ Copyright 2018, American Chemical Society. f) Viscosity as a function of shear rate for reducted and acylated lignin‐based resins. Reproduced with permission.^[^ ^234^ ^]^ Copyright 2021, MDPI. g) Synthetic route of DAL‐11ene and DAL‐12ane. h) Schematic diagram of the fabrication procedure of composites using DAL‐11ene and DAL‐12ane as photoinitiators and the mechanism of generation of free radicals in DAL under UV light. Reproduced with permission.^[^ ^235^ ^]^ Copyright 2020, American Chemical Society.

SLA is an additive manufacturing process that uses a movable platform, a UV light source to cure resin layer‐by‐layer, and a resin bath that is composed of photoinitiator, monomers, or oligomers, and other additives to build designed geometries. Although lignin has been recently reported to be capable of producing free radicals under natural light and shows a great potential as a photoinitiator, the low activity and poor stability of these free radicals highly limit the mechanical properties and performance of the products printed by SLA.^[^ ^228^ ^]^ Generally, lignin acts as an additive or is modified by structural design to produce a photoinitiator with high activity and strong stability for SLA printing. For example, materials of lignin‐containing CNC,^[^ ^229^ ^]^ kraft softwood lignin,^[^ ^230^ ^]^ and OSL^[^ ^231^ ^]^ have been used as additives to prepare nanocomposites via SLA printing. Lignin‐containing CNC can significantly decrease the *T*_g_ of methacrylate, which can reduce energy consumption during SLA printing (**Figure** **12**a). At low temperature, the *G*′ of lignin‐containing CNC/methacrylate nanocomposites decreases, but the reinforcing effect of lignin‐containing CNC becomes clear at high temperature due to the lignin‐containing CNC restricting the motion of methacrylate molecular chains. After post‐cure, the reinforcing effect of the *G*′ shows earlier than that before post cure, which attributes to the esterification between lignin‐containing CNC and methacrylate molecules (Figure 12b). In addition, the addition of softwood kraft lignin leads to higher exotherm enthalpy values, which helps to cure the resin at low temperature and decrease energy consumption. However, the unmodified lignin often shows an inefficient dispersion with methacrylate, which tends to settle to the bottom of the resin bath. The addition of unmodified lignin seriously hinders the curing process, and 1.0 wt% lignin addition results in more than 50% of the material without crosslinking during printing. Therefore, post‐cure by UV light is developed to ensure the full crosslinking of the non‐gel fraction of the printed 3D objects, yet further increases the time‐cost. OSL has also been reported to be blended with polyurethane for SLA printing, and different from the blend of softwood kraft lignin/methacrylate, the OSL improves its dispersion in polyurethane, thus enhancing the mechanical properties of the composite materials. However, the improvement of the mechanical strength of lignin materials fabricated by SLA is limited. Only a modest increase in tensile strength and elastic modulus with ≤1 wt% lignin addition, and excess lignin addition (1–3 wt%) instead causes a drastic reduction in performance of the printed composites. Therefore, efforts are made by chemical modification of lignin for stable SLA printing and good mechanical strength of the composites. Liu et al.^[^ ^232^ ^]^ used poly(ethylene glycol) (PEG) to modify lignin and then a photoreactive irgacure2959 was grafted onto the modified lignin to obtain a lignin‐based water‐soluble macromolecular photoinitiator (L‐PEG‐2959) through a coupling reaction (Figure 12c). The modified lignin has high light absorption within 200–400 nm wavelength range and is compatible well with glycidyl methacrylate‐modified gelatin. Under UV irradiation, a chemical cross‐linked lignin‐gelatin hybrid hydrogel is quickly formed by photopolymerization, and presents controllable swelling property, increased mechanical strength. Sutton et al.^[^ ^233^ ^]^ adopted the methacrylic anhydride modified OSL to blend with photopolymer resin and further demonstrated the printability for SLA. The blend displays Newtonian behavior with the viscosity ranging from 0.44 to 1.66 Pa·s, and the lignin content for smooth SLA printing reached 15 wt%. The modified lignin substantially improves the mechanical properties of the printed structures, yet the increased viscosities with lignin cause longer wait times for the recoating procedure and lead to a lower printing speed. The reduction and/or acylation used for lignin modification may provide an appropriate property to lignin (Figure 12d–f), which has no obvious effect on the viscosity of the blends, with all other resins exhibiting Newtonian behavior in the range of 0.4–0.6 Pa·s except the acrylic anhydride acylated one.^[^ ^234^ ^]^ To further broaden the responsive wavelength of lignin to light, Zhang et al.^[^ ^235^ ^]^ synthesized alkylated dealkaline lignins (DAL) through a facile one‐step esterification reaction between lignin and undecanoyl chloride or dodecanoyl chloride, and demonstrated that the modified lignin can be used as visible light macromolecular photoinitiator (Figure 12g). When combined with co‐initiator ethyl 4‐(dimethylamino) benzoate (EDAB), DAL‐undecanoyl chloride and DAL‐dodecanoyl chloride present excellent performance as macro photoinitiators in digital light processing 3D printing (Figure 12h). We anticipate that the two photoinitiators could also be used for SLA printing. In summary, although technical lignins without modification can be used as additives for SLA printing, the lignin content, and the enhanced performance of printed objects are limited. Modification of lignin is a promising strategy to obtain the value‐added lignin for SLA printing, and to highly improve the performance of printed objects. The abundant active sites of lignin provide a flexible way to meet the requirements for multifunctional materials.

## 5 Applications of Printed Lignin‐Based Materials

### 5.1 Bioactive Composites for Wound Dressing

**Figure 13:**
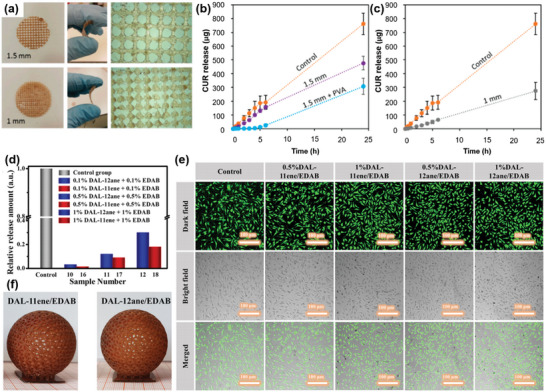
Printed lignin‐based bioactive composites for wound dressing. a) Digital images of the 3D printed lignin/PVA meshes. b) CUR release through b) 1.5 mm and c) 1 mm 3D printed meshes. Reproduced with permission.^[^ ^239^ ^]^ Copyright 2019, MDPI. d) Relative release amount of photoinitiators from the composites. e) Confocal laser scanning microscope images of Murine L929 fibroblasts incubated on polyethylene glycol diacrylate fabricated by using different photoinitiators and f) the printed hollow spheres of DAL‐11ene/ethyl 4‐(dimethylamino) benzoate (EDAB) and DAL‐12ane/EDAB‐based composites. Reproduced with permission.^[^ ^235^ ^]^ Copyright 2020, American Chemical Society.

Lignin is a versatile biopolymer that features intrinsic antioxidant, antibacterial, anti‐UV, antitumor, and antivirus activities, which make lignin a promising bioactive material.^[^ ^19^, ^46^, ^236^ ^]^ Bioactive materials are extremely beneficial for healthcare applications such as wound dressing and tissue engineering. In recent years, lignin nanocomposites have been used as potential alternatives in drug and gene delivery for disease therapy. With adaptability for biomedical applications, the 3D printing of lignin allows the construction of customizable and personalized objects that can be used as biomaterials.^[^ ^237^ ^]^ Oveissi et al.^[^ ^238^ ^]^ used lignin to crosslink hydrophilic polyether‐based polyurethane, and the formed composite hydrogel was printed by DIW and demonstrated to be biocompatible with human dermal fibroblasts. The hydrogel is also capable of supporting cell growth and has no obvious effect on cell viability, and shows great potential in biomedicine. The lignin composites fabricated by FFF can also be used for wound dressings, as demonstrated by Domínguez‐Robles et al.,^[^ ^239^ ^]^ the lignin/PLA meshes containing drugs were printed by FFF, and the drug can easily diffuse through the mesh pores to the wound (**Figure** **13**a). Interestingly, the presence of lignin provided a slower curcumin release than the control one, and the incorporation with PVA further delayed curcumin release (Figure 13b,c). With antioxidant properties, lignin contributes to reducing the concentration of the reactive oxygen species that are strongly linked to the pathogenesis of chronic wounds, which makes lignin/PLA a green and low‐cost 3D printable biomaterial with efficient effect in wound treatment. The lignin composites with controlled‐release property can be customized by SLA or digital light printing. As the printed DAL‐11ene and DAL‐12ane mentioned above, the release of the two polymeric tablets photoinitiated by DAL‐11ene/EDAB and DAL‐12ane/EDAB in methanol decreased compared with that of Irgacure2959 (Figure 13d).^[^ ^235^ ^]^ The authors also evaluated the biosafety of polymeric tablets by live/dead staining, and the results demonstrated that L929 cells proliferated and grew very well on the surface of tablets, with the density of cells much higher than the control one (Figure 13e). With the DAL‐11ene or DAL‐12ane concentration increase, no obvious difference was observed, indicating excellent biosafety of the polymeric tablets. The printed 3D structures displayed high resolution and good edge definition (Figure 13f), suggesting that the printed composites have potential in packaging and environmentally‐friendly photopolymerization materials.

### 5.2 Array Composites for Photoelectrochemical Hydrogen Evolution

**Figure 14:**
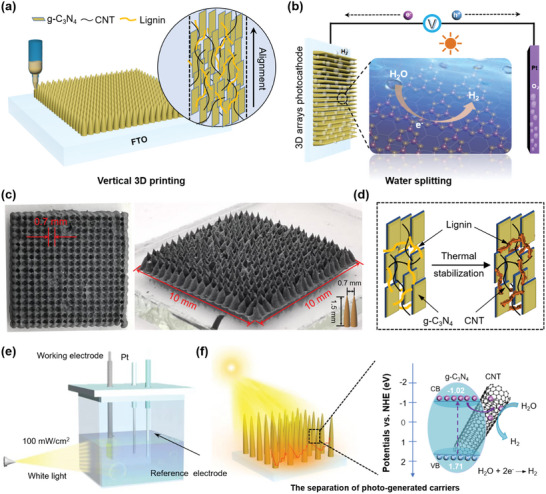
Printed structural composites for photoelectrochemical hydrogen evolution application. a) Vertical 3D printing of g‐C_3_N_4_/CNT/lignin on a conductive substrate and its micro‐structure. b) Schematic of the photoelectrochemical water splitting by using the printed arrays as the photocathode. c) Images of the printed arrays from top and side view. d) Schematic to show the interaction among compositions in printed arrays after thermal stabilization and e) the photoelectrochemical measurement under 100 mW cm^−2^ light, as well as f) the photoelectrochemical mechanism for the printed arrays. Reproduced with permission.^[^ ^214^ ^]^ Copyright 2019, Wiley‐VCH.

DIW is a promising technique for the alignment of 2D planar nanomaterials, such as the semiconductors of g‐C_3_N_4_ and MXenes, offering significantly improved performance to these materials. However, the application of such printed materials is limited in liquid reactions due to the weak interaction between the 2D planar structures. In natural wood, lignin acts as a binder contributing to the wood strength, water stability, and rigidity of the cell walls, and also enables to improve the mechanical properties and stability of other materials.^[^ ^6^, ^240^ ^]^ By incorporating of the adhesive property of lignin with semiconductors, the structural composites constructed by 3D printing would highly broaden the potential applications of lignin, especially the stable structures in liquid reactions. For example, Jiang et al.^[^ ^214^ ^]^ demonstrated vertically 3D printed forest‐like lignin/g‐C_3_N_4_/CNT arrays with enhanced photoelectrochemical hydrogen evolution performance (**Figure** **14**a–d). The multiple scattering of the arrays elongates the incident light path, meanwhile, the CNT further promotes photoelectron‐hole pair effective separation (Figure 14e,f), thus improving the utilization of light and the transfer efficiency of photoelectrons. The inherent hydrophobicity and good adhesivity of lignin endow the printed arrays with high durability, and enable them to continuously produce hydrogen. More importantly, lignin is an aromatic biopolymer, and a donor–acceptor conjugated structure is considered to be formed due to the intermolecular *π*–*π* interaction between lignin and g‐C_3_N_4_, resulting in an enhanced photoelectrochemical hydrogen evolution performance. The authors also demonstrated that lignin as a natural binder is much superior to other binders of PVA and PEG, which may be because their non‐conductive and long chain molecular structures impede the generation and separation of photoelectrons. With the world's over‐consumption of fossil‐fuels, such materials using green and renewable biomass as a composition will open a new avenue for sustainable production of clean energy.

### 5.3 Carbon‐Based Composites for Electronics and Environmental Remediation

**Figure 15:**
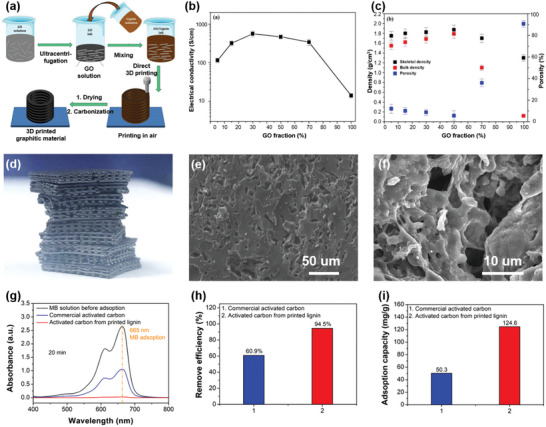
Printed lignin‐based carbon‐based composites for electronics and environmental remediation. a) Schematic of the fabrication process of DIW printed lignin/GO‐based carbon materials. Evolution of b) electrical conductivity and c) density and porosity of various lignin/GO‐based carbon materials. Reproduced with permission.^[^ ^213^ ^]^ Copyright 2020, Elsevier. d) Image of activated carbon from the printed lignin scaffold. e,f) Microstructure of the printed activated carbon. Comparison of the g) UV–vis spectra, h) removal efficiency of the methylene blue solutions after 20 min adsorption, and i) adsorption capacity of the commercial and printed activated carbon. Reproduced with permission.^[^ ^207^ ^]^ Copyright 2020, Wiley‐VCH.

In the development of low‐cost, high‐performance materials, carbon‐based materials play a pivotal role in energy storage, heterogeneous catalysis, environmental remediation, and soil additives, etc.^[^ ^241^, ^242^, ^243^, ^244^, ^245^, ^246^, ^247^ ^]^ Lignin as an abundant and renewable aromatic compound shows the highest carbon content (>60%) in lignocellulose, making it a promising precursor for the elaboration of carbon‐based materials. Recently, lignin has been widely used for production of active carbon,^[^ ^248^, ^249^, ^250^ ^]^ carbon quantum dots,^[^ ^251^, ^252^, ^253^ ^]^ carbon fiber,^[^ ^254^, ^255^, ^256^, ^257^, ^258^ ^]^ etc. Although few works have been performed to construct lignin‐based carbon materials using 3D printing, the printed lignin‐based composites exhibit unique properties and excellent performance in electronics and environmental remediation. For example, graphitic structures with tunable density were demonstrated that can be sharp in 3D by DIW of lignin/graphene oxide (GO) inks and subsequent carbonization (**Figure** **15**a).^[^ ^213^ ^]^ By adjusting the lignin fraction, the density and graphitic order can be tailored as required, which leads to unique electrical and mechanical properties. The conductivity of graphene oxide is only 14 S cm^−1^, and that of lignin/GO with a formulation of 70/30 increases up to 560.5 S cm^−1^ (Figure 15b). The bulk density and pore volume of carbonized 3D‐printed structures can be adjusted in a wide range of 0.2–1.8 g cm^−3^ and 0.024–0.12 cm^3^ g^−1^, respectively (Figure 15c). The tunable electrical and mechanical properties make lignin‐based carbon materials promising candidates for a broader range of applications, such as supercapacitors and batteries. In addition to the application in electronics, the carbon materials based on 3D printing of lignin also display an attractive candidate in environmental remediation. Active carbon as a common material is widely used for the adsorption of dyes, organics, toxic gases, and metals. By incorporating 3D printing, the printed lignin‐based active carbon materials have been demonstrated to possess a much higher adsorption efficiency than commercial carbon materials (Figure 15d–i).^[^ ^207^ ^]^ First, during carbonization and activation process, the dense structure of printed lignin filaments becomes porous, and the micropores in the printed filaments are important for improving the mass transfer efficiency. Second, the space between the adjacent filaments is also tunable, which results in a hierarchical porous structure in the product. Third, the active carbon with 3D scaffolds also enables multiple recycling, which is desirable for the practical treatment of industrial pollutants.

### 5.4 Engineering Materials

**Figure 16:**
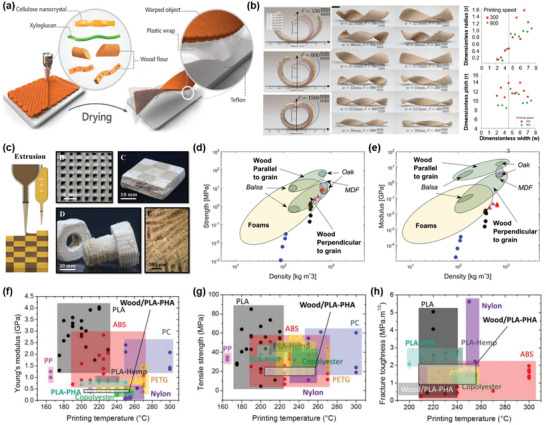
Printed wood/lignin‐based composites for engineering applications. a) Schematically showing the wood warping composite by 3D printing, including the printing and drying process. b) The 3D warped objects at different velocities and the dimensionless radius or pitch versus dimensionless width. Reproduced with permission.^[^ ^266^ ^]^ Copyright 2022, MDPI. c) Schematic and images of the multimaterial extrusion‐based DIW printing of a mashrabiya, a chess board model, a nut, screw, and the cross‐section SEM of printed material. d) Strength and e) modulus of printed composites compared with natural woods. Reproduced with permission.^[^ ^261^ ^]^ Copyright 2019, Wiley‐VCH. f) Young's modulus, g) tensile strength, and h) fracture toughness of wood‐based filaments printed by FFF. Reproduced with permission.^[^ ^260^ ^]^ Copyright 2019, MDPI.

Lignin is a structural component in wood and adds strength and rigidity to the cell walls. It can even regulate the transport of water and solutes in plants and protect carbohydrates from phytopathogens and other environmental stresses.^[^ ^18^, ^258^ ^]^ Therefore, wood also acts as a lignin‐based material. In recent years, the combination of wood flour with 3D printing has raised increasing concerns.^[^ ^259^, ^260^, ^261^, ^262^, ^263^, ^264^ ^]^ With appropriate additives or binders, wood flour can be printed into many complex engineering products, such as interlocking products, crate, napkin ring, fully‐functional treasure chest.^[^ ^265^ ^]^ These complex and functional products are difficult to prepare by traditional woodworking methods. 3D printing is self‐replicating technique that can be extended to wood. For example, softwood tracheids or more complex structures can be formed by the 3D printing of wood flour, which offers a unique microstructure and functions to the materials.^[^ ^264^ ^]^ Wood warping is a common phenomenon due to the changes of moisture content in the anisotropic wood structure. Such objects can also be fabricated by 3D printing. Kam et al.^[^ ^266^ ^]^ developed a printable ink composed of 100% wood‐based materials (**Figure** **16**a, wood flour, CNC, and xyloglucan). By carefully controlling the flow rate and printing path, the morphology of the fully dried wooden objects can be tuned from plate to annulus due to the anisotropic shrinkages (Figure 16b), which opens an avenue toward the 4D printing of wood. The group further demonstrated that printable ink is suitable for multimaterial printing (Figure 16c), and physical properties involved strength and modulus of the “printed wood” are similar to those of natural timber (Figure 16d,e).^[^ ^261^ ^]^ The wood flour can also be used for FFF printing by blending with plastic polymers. Guessasma et al.^[^ ^260^ ^]^ conducted the wood‐PLA/polyhydroxyalkanoates printing with the printing temperature between 195 and 220 °C. The printed wood‐based filament shows comparable stiffness (Young's modulus) with ABS, polyethylene terephthalate, nylon, et al. (Figure 16f). However, the tensile strength and fracture toughness of wood‐based filament are limited compared with those plastic polymers (Figure 16g,h), which may be due to the inhomogeneity of filament components. Despite the limitation, the 3D printing of wood flour offers the objects unique properties that can overcome the imperfections of traditional wood. In addition, the wood flour can also be blended with other polymers^[^ ^267^, ^268^, ^269^ ^]^ to improve the performance of the printed objects, and lignin in the materials will play a role similar to that in natural wood.

### 5.5 Other Potential Applications

Lignin features natural biological activities, biocompatibility, nano‐scale adjustability, flexibility in structural modification, hydrophilicity, and hydrophobicity,^[^ ^10^, ^11^, ^12^, ^15^, ^19^, ^20^, ^21^ ^]^ which can be tailored into many functional materials for other potential applications. It has been reported that cellulose electronegativity is an attractive candidate for efficient and tunable ion regulation devices, and its surface charge can be easily tuned by chemical modification.^[^ ^270^, ^271^ ^]^ It has been demonstrated that lignin is also an effective composition of nanofluidic composite membranes for efficient ion transport and osmotic energy harvesting.^[^ ^272^, ^273^ ^]^ For example, it was fund that lignin can not only significantly facilitate the dispersion of the lignin in the sulfonated PEEK (SPEEK) matrix, but also highly improve the proton conductivity and ion selectivity of the SPEEK/lignin membrane, making the composite membrane a promising candidate for the next generation larger‐scale vanadium redox flow batteries.^[^ ^272^ ^]^

The nanostructure of lignin does not significantly affect the changes of lignin functional groups, and shows a large specific surface area, providing better structural features and biological activities to lignin than the original one.^[^ ^19^ ^]^ The nanoscale lignin can be printed to nanocapsule or other composites that act as controlled release drugs. Lignin nanoparticles can be further physically tuned and chemically modified, giving the composites great potential in antibacterial, catalysis, and even bio‐photonic materials.^[^ ^274^, ^275^ ^]^

Recently, the mechanically strong and tough nanocomposites have also gradually developed into ideal materials for future engineering and functional applications.^[^ ^276^, ^277^, ^278^ ^]^ Such materials are indispensable in our daily life, and are widely used in biomedical load‐bearing materials, armor, foldable electronics, etc. With abundant functional groups, lignin is a promising candidate for the fabrication of nanocomposites that have both high strength and toughness.^[^ ^277^, ^279^ ^]^ Combined with 3D printing to composites with designed patterns, lignin shows great potential in engineering and biomedical load‐bearing materials, such as ligament, cartilage, and cell culture scaffold.

Lignin, with aromatic properties and high carbon content, can be directly used to prepare graphene‐like structures for energy‐related applications. Ye et al.^[^ ^280^ ^]^ developed a facile approach to transform wood surfaces into hierarchically porous graphene using CO_2_ laser scribing. The graphene patterned on wood surfaces is mainly from lignin, and allows the resulting material to be used in various high‐performance devices, such as green energy evolution electrodes and supercapacitors for energy storage. Zhang et al.^[^ ^281^ ^]^ further demonstrated that lignin films can be directly transformed into graphene electrodes by CO_2_ laser irradiation, which exhibit good electrochemical performance. Inspired by this approach, 3D printed lignin‐based materials can also be treated by a CO_2_ laser, which may facilitate the applications of lignin in supercapacitor, sensors, flexible electronics, and even smart materials. In summary, the development of lignin 3D printing and lignin functional materials is an important point toward future directions in advanced materials.

## 6 Concluding Remarks

Extensive research has revealed that the structure‐rheology relationship of lignin does an important effect on the printability of lignin/polymer blend inks and the performance of the resulting composites. Although the 3D printing of lignin is compatible with FFF, DIW, SLA, and SLS techniques, the good printability does not mean that lignin can be directly printed into 3D structures with high stability, desired property, and performance. Additives, especially macromolecule with linear structure, are essential for successfully constructing lignin‐based 3D objects. FFF printing of lignin is highly controlled by its molecular structure and molecular weight, organosolv hardwood lignin and oligomeric kraft softwood lignin as mentioned above are common feedstocks for FFF that have flexible segments and low molecular weight. DIW is suitable for the 3D printing of lignin due to the tunable rheological behavior, and not only allows the alignment of compositions but also enables the vertical 3D printing. Structure and type of lignins appear to have no significant influence on the printing process. DIW are either extrusion‐based or meniscus‐guided, and enables to construct diverse functional 3D architectures in micro‐ and nanometer scale. As an aromatic biopolymer with abundant active sites, lignin can be used for SLA printing by coupling with photoinitiators. The photopolymers in the printable blends play a decisive role in the process of SLA printing. With powder morphology and transition temperature, lignin is also compatible with the SLS technique but still presents a limitation in understanding the structure‐rheology relationship.

Traditionally, the utilization of lignin is mainly based on three ways: I) Direct use, such as antioxidants, antibacterial agents, stabilizers, binders, food additives, etc., II) modification or depolymerization for materials or chemicals, including thermoplastics, gels, nanomaterials, fuels, etc., III) carbon‐based materials, such as activated carbon, carbon fiber, carbon quantum dots, etc. The utilization of traditional lignin‐based materials has mainly applied its “passive” functionality, that is, frequently‐mentioned bioactivities, additives, or binders, yet with poor stability, and limited property and performance. The immiscible and brittle natures of original and technical lignins impede its wide application. Although modification or depolymerization is believed to be an effective way to add value to lignin and improve its interfacial compatibility with other polymers, not so much about taking full advantage of its specific properties, such as the redox and nutrition regulation characteristics. And the current strategies for modification or depolymerization are confronted with great challenges in focusing on clear‐cut methods using green‐chemistry and low‐cost techniques. The microporous structure is an important parameter for carbon‐based materials, which has a decisive influence on the charge (electron/ion) transport capacity. Unfortunately, the out‐of‐order microporous structure of carbon‐based materials from lignin prepared through conventional solution processing results in the formation of tortuous charge transport paths, which deteriorates the performance in energy storage and conversion.

Combined with 3D printing techniques, lignin is expected to act an active functional material for added value, which has been demonstrated to be feasible for lignin‐based materials through additive manufacturing. The development of lignin‐based polymers requires desired chemical and physical properties coupled with improved 3D printing techniques. Although 3D printing techniques are becoming mature and have given new insight and opportunities for the use of lignin, challenges and limitations still need to be focused on and overcome. First, 3D printing of lignin relies too much on the addition of petroleum‐based polymers due to the requirement of improved rheological behavior, such as viscoelasticity, modulus, and melting point. One solution is the replacement of petroleum‐based feedstocks with other green polymers to construct fully biodegradable materials, but more research is needed to make the inks adapt to 3D printing. Second, 3D printing with multiple materials and multi‐scale is limited. Materials for tissue engineering and robotic applications generally require multiple functional compositions, yet multiple materials usually lead to poor rheological behavior for specific 3D printing technique. Hybrid additive manufacturing will be a trend to meet the requirements for various functions. Third, a high 3D printing resolution is needed for lignin. Due to the complexity of lignin structure and morphology, the printing processes of lignin nanocomposites often generate flaws inside the printed objects, leading to deteriorated mechanical properties. For example, due to the unsatisfied bonding between layers, the shape of lignin powder can easily create voids in printed products. On the other hand, lignin filaments extruded by FFF and DIW are usually tough and thick, resulting in a low‐quality product.

Significant progress in additive manufacturing through the extensive research has revealed many fundamental challenges and demands for further research. For example, additives capable of cross‐linking with lignin or forming a network through in situ polymerization during 3D printing are highly desirable. Such chemically active additives that may participate or catalyze the cross link of lignin polymers will simultaneously affect the rheology and the morphology, inducing an appropriate viscoelasticity and a high 3D printing resolution for 3D printing. Additives may also modify the surface chemical or physical properties of lignin, such as making it hydrophobic or hydrophilic, thus enhancing the interfacial compatibility and adhesion between adjacent printed layers. Catalyzing or stimulation of such in situ polymerization during the printing process might be needed through some external inputs, such as laser or active gases. Equally desirable is the post printing treatment to promote the microstructure evolution and to eliminate residual voids and inhomogeneity. Surface tension or surface energy driven molecular segregation during the additive manufacturing and its impacts on microstructure and properties and performance of the 3D structure has not been studied. Incorporation of inorganic components in additive manufacturing of lignin‐based materials remains largely unexplored territory. In situ monitoring as well as post‐inspection are essential for the further development of high quality of additive manufacturing of lignin‐based materials.

3D printing techniques will continue to move toward the developments and directions of the diversification of materials, structures, and methods, and is highly compatible with lignin. Structural properties and functions of lignin still need to be further developed to meet the demands in biomedicine, aerospace, and automobile applications. We anticipate that the development of additive manufacturing will largely broaden the value‐added applications of technical lignins, and promote the sustainable utilization of lignin “from nature to nature.”

## Conflict of Interest

The authors declare no conflict of interest.
